# Supplementary material for: Towards a bottom-up understanding of antimicrobial use and resistance on the farm: A knowledge, attitudes, and practices survey across livestock systems in five African countries
Source: PLoS One. 2020 Jan 24;15(1):e0220274. doi: 10.1371/journal.pone.0220274 (PMC6980545; doi:10.1371/journal.pone.0220274)
Supplement: S1 Text — (DOCX) [file pone.0220274.s001.docx]

**S1 Appendix. Study location, Sampling Strategies and Supplemental Tables**

**1. Ghana**

The KAP study was carried out in Dormaa Central Municipal of Brong Ahafo Region, Ghana. It is one of the 260 Metropolitan, Municipal and District Assemblies (MMDAs) in Ghana, and forms part of the 12 of Municipalities and Districts in the Bono Region. The municipality is found in the semi-equatorial zone with bi-modal rainfall pattern. It lies within longitudes 3^0^ West and 3^0^ 30’ West and latitudes 7^0^ North and 7^0^ 30’ North. The municipality has a total land area of 1,210.28square kilometres, which is about three (3.1) percent of the total land area of Brong Ahafo Region.

The population of the Municipality according to 2010 population and housing census stands at 50,871 with 23,970 males 26,901 females.  It is bound in the north by the Jaman South Municipal and in the east by the Dormaa East District, in the south and south-east by Asunafo North Municipal and Asutifi North District, in the west and south-west by Dormaa West and in the west and north-west by La Cote d’Ivoire.

About 68.4% of households in the municipality are engaged in agriculture (21). It is the highest commercial poultry producing district in the country with about 3.1 million layers and 1 million broilers. The farmers in the district are served by government veterinary staff including 1 District Veterinary Officer (DVO) and 5 Veterinary Technical Officers (VTOs).

**Figure 1. Map of the study area in Ghana.** See map legend for description of map markers. Maps were created using ArcGIS software by Esri. The base map is sourced from Esri and modified in ArGIS Pro. "Light Gray Canvas" [basemap] https://www.arcgis.com/home/item.html?id=ee8678f599f64ec0a8ffbfd5c429c896. The administrative later was sourced from MapLibrary <http://www.maplibrary.org/library/stacks/Africa/index.htm> Dec 13th, 2018.

**
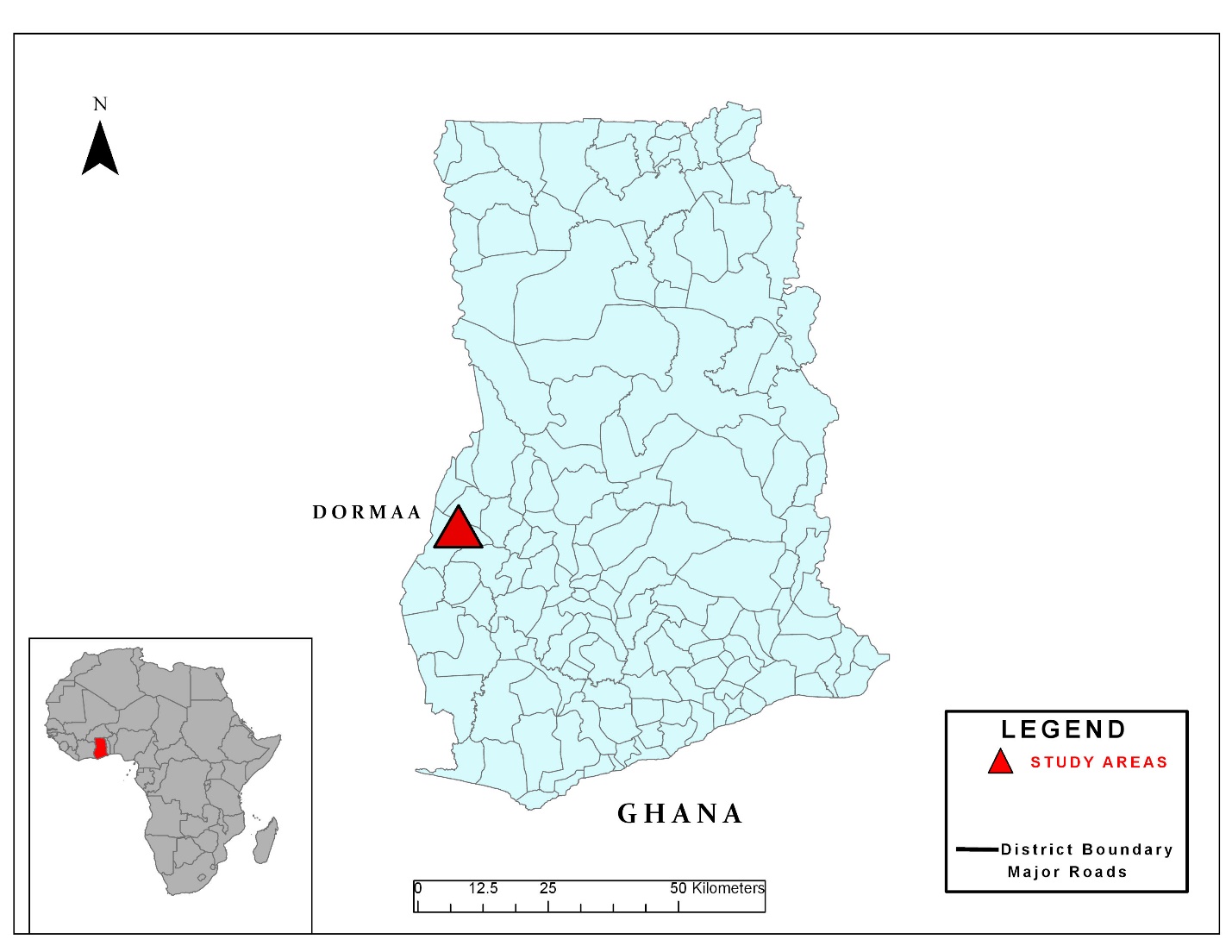
**

## KAP Survey Development

Focus group discussions were conducted among two groups of farmers (between 8 -12 people) and among veterinary officers in the municipality. Seven key informant interviews were also conducted among the following stakeholders: drug dealers (2), feed millers (2), quasi vet technician (1), and veterinary staff (2). Thematic analysis of qualitative interviews was used to develop a KAP survey instrument of over 200 items that included a broad range of demographics, livelihood, health, hygiene and biosecurity topics relating to factors that could promote AMU, sub-optimal AMU practices, and AMR (see Appendix I for KAP questionnaire). KAP surveys were administered by a group of four local research assistants using tablets with the Kobo Collect® application. Assistants were trained in Dormaa at a four-day training and KAP piloting event. The survey was administered in English and Twi, with the respondent indicating what language they were most comfortable in using. Interviews lasted around 1 hour. All informants provided consent to participate through signature or thumbprint. See Appendix I for FGD and KII topics for animal healthcare professionals and farmers. See Appendix II for Informed Consent forms.

**Sampling Procedure**

Census records with production data were not available for Dormaa so a snowball sampling approach was used whereby veterinary officers were initially contacted to provide names of individuals currently engaged in layer production. Recalled layer farmers were then consulted during the KAP survey to provide names of individuals who were currently engaged in layer production. This process was conducted until the data collection period had ended.

## Ethical Approval

Permission to conduct the study was sought and obtained from the Ministry of Health Ethical Review Board (ID No. 014/10/18), Ghana. Letters of introduction, which was followed by formal verbal explanation of the study, were submitted to the poultry farmers’ association in the district. Every participant involved in this study provided informed consent either through written or verbal means prior to providing any information (FGD, KII, KAP Survey, and Intervention). The respondents were allowed to withdraw from the study at any point without penalty. In addition, they were told that all their responses will be kept completely confidential.

**2. Kenya**

The study was conducted among 76 layer farmers in Gatundu North Subcounty, Kiambu County of Kenya (see Figure 2) between December 2018 and March 2019. The economy of Kiambu County is dominated by agriculture with 75% of the population under small-scale production system [1]. Major economic activities include livestock production (dairy, sheep, goats, pigs and poultry), crop production (for example, coffee, tea, and horticulture), small-scale trading (selling vegetables/fruits) and, to a lesser extent, large-scale businesses (e.g., real estate entrepreneurship).

**Figure 2. Map of the study area in Kenya.** See map legend for description of map markers. Maps were created using ArcGIS software by Esri. The base map is sourced from Esri and modified in ArGIS Pro. "Light Gray Canvas" [basemap] https://www.arcgis.com/home/item.html?id=ee8678f599f64ec0a8ffbfd5c429c896.. The administrative later was sourced from MapLibrary <http://www.maplibrary.org/library/stacks/Africa/index.htm> Dec 13th, 2018.


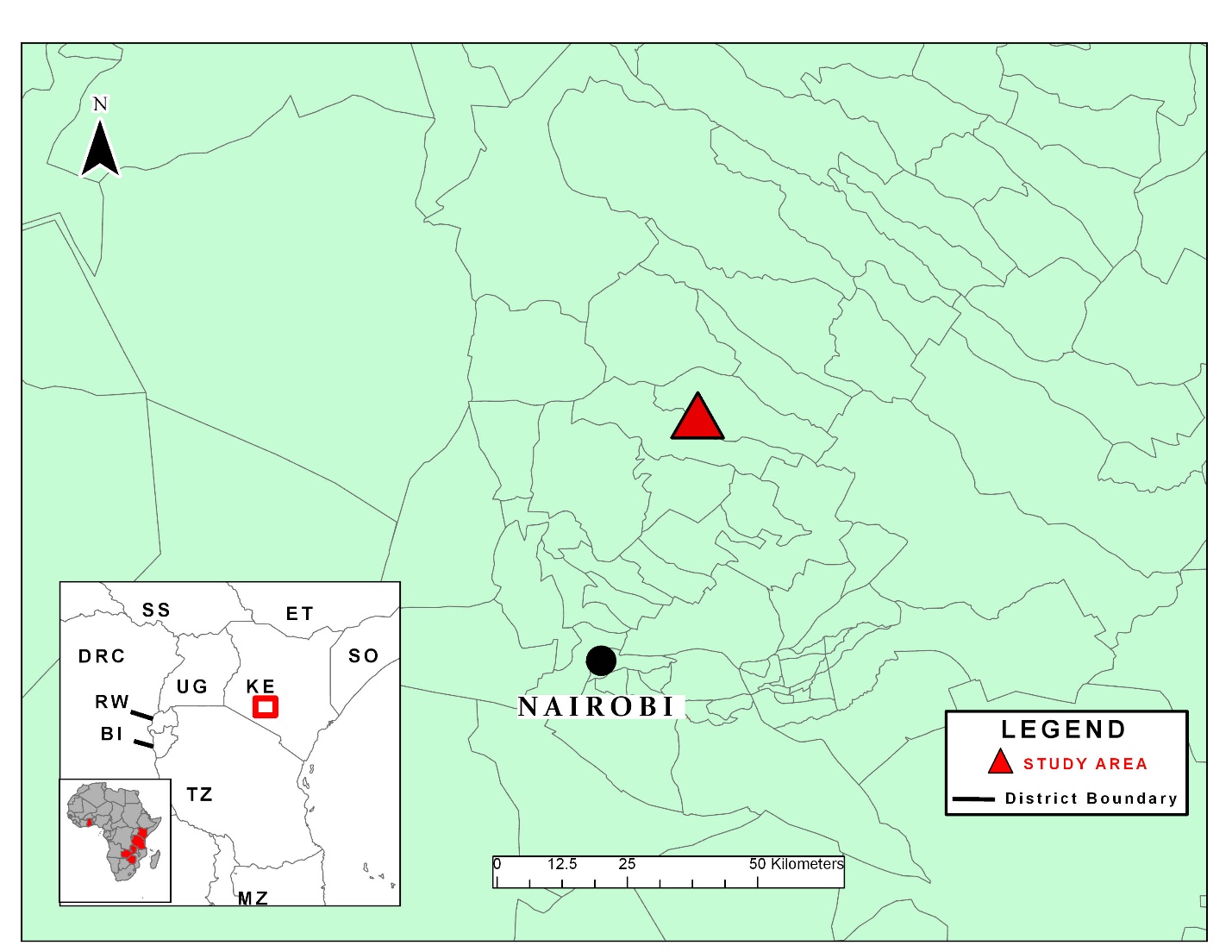


According to the Kenya Government annual livestock production data, Kiambu County has the largest poultry and layer production systems in the country (see Figure 3) with approximately 1,068,873 layers and 622,430 broilers (25.7% and 20.4% of the country total, respectively). Gatundu North Subcounty, where this study was conducted, has the highest number of layers consisting of approximately 241,500 layers including 60,000 layers in Mang’u Ward, 163,500 in Chania Ward, 10,000 in Gituamba Ward, and 8,000 in Githobokoni Ward. Prior research conducted in Kiambu County demonstrates that antibiotics are widely used by farmers and acquired without prescription from agrovet shops [1]. In addition, high prevalence of AMR in poultry have been described in the country in areas with production systems similar to Gatundu North [2].

**Figure 3**. **The proportion of households investing in poultry production by poultry type.** The red arrow denotes the study area.


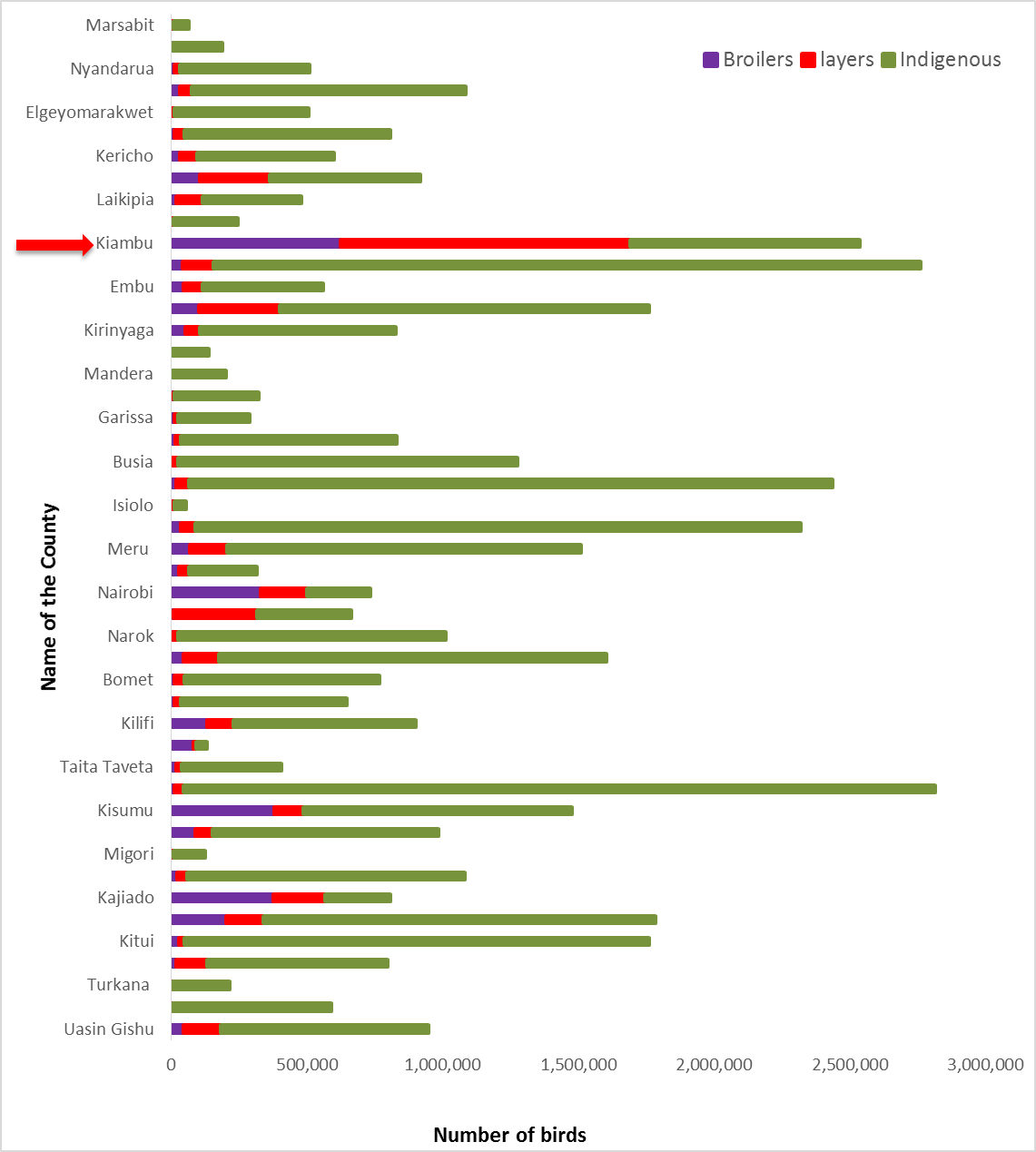


## KAP Survey Development

An interdisciplinary research team comprised of animal health experts and social scientists from the FAO, the Ministry of Agriculture, Livestock, Fisheries and Irrigation (MALFI), and Sub County animal healthcare workers used a mixed-methods approach to develop the KAP survey. This included conducting six mixed-gender FGDs with farmers, six KIIs with agrovets, and six KIIs with Sub County veterinary officers and assistant veterinary officers. FGDs and KIIs were concentrated around twelve major themes relating to AMU and AMR including farm management and economic practices, disease histories, knowledge, attitudes, and practices relating to AMU and AMR, including use, governance, regulations, policies, and enforcement.

Thematic analysis of qualitative interviews was used to develop a KAP survey instrument of over 200 items that included a broad range of demographics, livelihood, health, hygiene and biosecurity topics relating to factors that could promote AMU, sub-optimal AMU practices, and AMR. KAP surveys were administered by a group of four local research assistants using tablets with the Kobo Collect^®^ application. Assistants were taken through a comprehensive four-day training and KAP piloting. The survey was administered in English, Kiswahili or Kikuyu, with the respondent indicating what language they were most comfortable in using. Interviews lasted around one hour. All informants provided consent to participate through signature or thumbprint.

**Sampling Procedure**

Census records with production data were not available for Gatundu North so enumerators conducted a door-to-door survey within their local neighbourhoods. A random selection of households was selected from these lists and provided to enumerators. This sampling was not exhaustive of all layer farmers in Gatundu North as enumerators had two to three days for door-to-door surveys. As enumerators were on foot, this biased the sample to farms close to the homes of enumerators.

## Ethical Approval

This study was approved by the AMREF Health Africa Ethics and Scientific Review Committee (AMREF-ESRC P551/2018). This research was also approved by the Institutional Animal Care and Use Committee of KALRO-Veterinary Science Research Institute, Muguga upon compliance with all provision vetted under and coded: KALRO-VSRI/IACUC016/28092018. Prior to requesting for consent, an information sheet (IS) containing a detailed narrative of the study and its aims was provided to potential participants who could read, and was read out to those who could not. Participants were informed of the research purposes including the benefits and risks of participation. The respondents were assured of their right to withdraw from study participation at any point, and necessary precautions were made to insure and maintain confidentiality, anonymity and voluntarism throughout the study. A written informed consent was sought from all study participants who could write. For those who could not write a thumbprint signature was requested.

# 3. Tanzania

The cross-sectional study was undertaken in Maasai homes and in markets within Longido District, Arusha Region in northern Tanzania between November 18^th^- 4^th^ December 2018 (see Figure 4). Four wards within Longido District were targeted for sampling, including: 1) Longido, 2) Isinya, 3) Gilailumbwa, and, 4) Engarenaibor. These locations were purposefully selected in the field after taking into consideration social economic activities and distances between each ward. Longido ward is where the District headquarters is located, and the main road from Arusha City to Namanga Border (between Tanzania and Kenya) runs through Longido district. Longido is also the center of most businesses in the district and offers reliable transportation to the urban center of Arusha. Isinya is located 30 kms from Longido district headquarters overlooking the slopes of Mount Kilimanjaro, and adjacent to a wildlife management area. Gelailumbwa is the furthest of all wards (55 kilometers) from Longido district headquarters bordering Ngorongoro district to the northeast. Very little crop cultivation takes place in the area. Engarinaibor is a mixed economy ward with small holder cultivation of crops taking place compared to the rest. It is also home to the famous mining village of Mundarara where ruby has been mined for a number of years.

**Figure 4. Map of Study Area**. See map legend for description of map markers. Maps were created using ArcGIS software by Esri. The base map is sourced from Esri and modified in ArGIS Pro. "Light Gray Canvas" [basemap] https://www.arcgis.com/home/item.html?id=ee8678f599f64ec0a8ffbfd5c429c896.. The administrative later was sourced from MapLibrary <http://www.maplibrary.org/library/stacks/Africa/index.htm> Dec 13th, 2018.


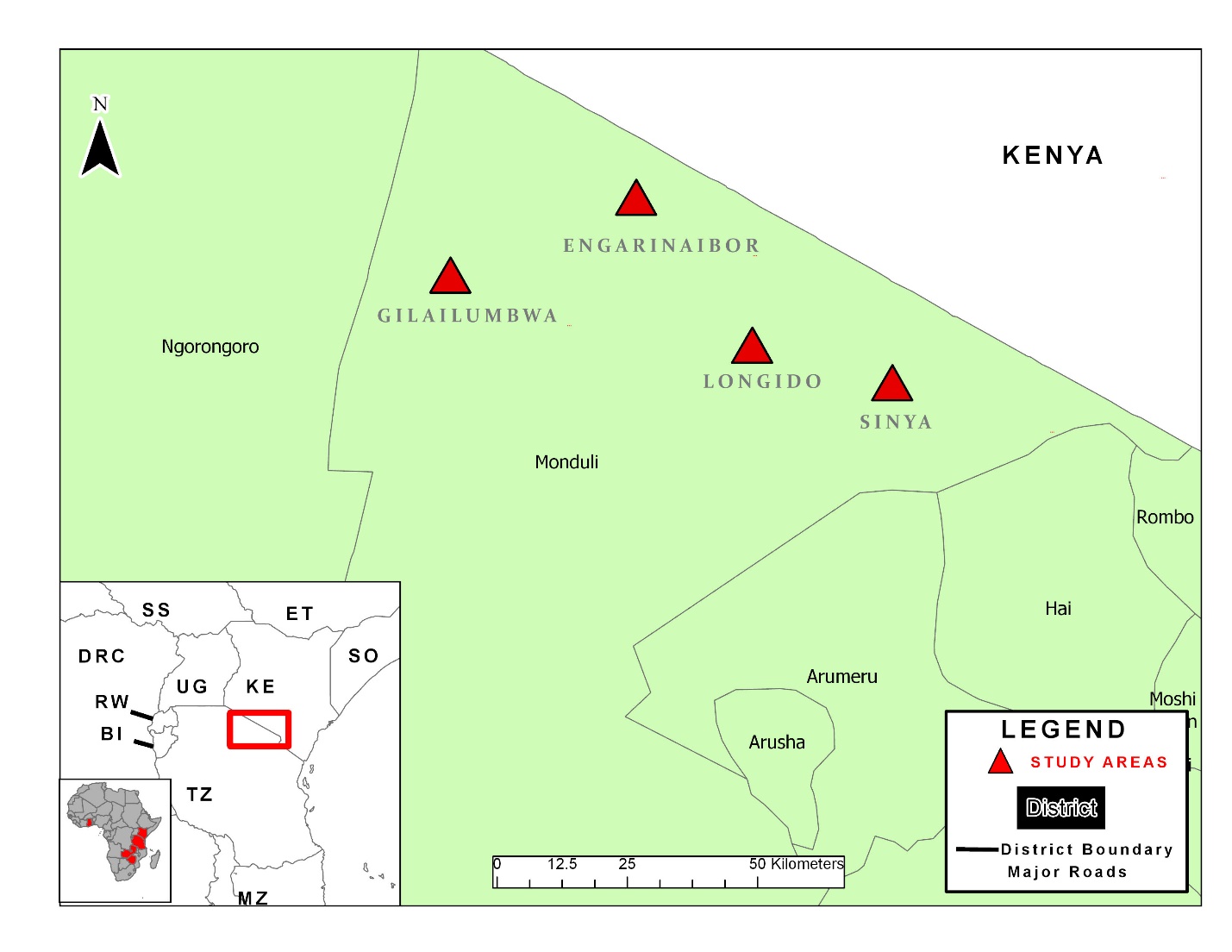


**Study Population**

The Maasai and related *Maa*-speaking pastoralists are found throughout Tanzania and Kenya. Traditionally Maasai were nomadic pastoralists who moved with their herds in search of grass and water. Today most Maasai are agro-pastoralists who grow some crops (mostly maize and beans), although livestock products still provide the majority of energy [3, 4]. Most Maasai inhabit rural areas within extended family compounds called *bomas* (*enkang* in *Maa*) organized along patrilineal lines. Polygamy remains common, especially in rural areas, and marriages are structured by patrilocal residence, clan exogamy, and bride price, often paid in livestock, is required. Beginning in early adolescence, the division of labor becomes gender specific with females milking cows and preparing family meals. Children begin tending livestock by the ages of 5-6, starting with kids/calves, which stay around the boma, while boys progress to herding adult goats and cattle as they age and become *moran* (warriors) [5–7] Older men spend their time tending to livestock mainly through overseeing the young herders and in business ventures. Some Maasai men, including those in our study sites, have found economic success working in the Tanzanite mines, a gem which can only be found in northern Tanzania, and have built cinderblock houses and pit toilets within their compounds. For Longido Maasai, popular mining sites are those found in Mundarara village located in Engerinaibor ward. A small minority has also purchased vehicles and constructed guesthouses and hotels in and out of the district. Other men have pursued wage-labor jobs, often working as security guards for banks and safari companies in Arusha, a common livelihood strategy within the Maasai [8–10]

Across Longido District, the average annual rainfall is around 500mm and so designated as a semi-arid ecological zone. Rainfall is bimodal with short rains falling between November and January and long rains between March and May. In more recent times, however, the rains are becoming much less predictable and the short-rains often fail entirely [11]. Vegetation is dominated by woody shrubs and trees particularly *Acacia tortilis, Acacia nilotica* and *Commiphora africana* common throughout the area. Soils are mostly red clay and light sandy soils [12]. With the exception of Mount Longido (≈elevation 2637 m), the elevation ranges between 1400-1600 meters above sea level.

KAP Survey Development

An interdisciplinary research team comprised of animal health experts and social scientists from the FAO, the Ministry of Livestock and Fisheries (Veterinary Service Division) and National Institute for Medical Research, used a mixed-methods approach to develop the KAP survey. The study employed a slightly modified version of the exploratory cross-sectional survey design approached [13] using both qualitative and qualitative data to inform quantitative parameters and confirm quantitative measures. Qualitative data collection included six FGDs with livestock producers and consumers of livestock products, who were mostly heads of households and were conveniently selected. In addition, “Opinion Shapers”, including village leaders, traditional elders, political leaders formed another group category of participants for FGD. Participants in FGDs were identified by sending out information about the study to the ward office two days before the FGD. A message was then transmitted to village leaders who invited participants from their village willing to sit and discuss general issues of livelihood and subsistence. Influential people were purposefully identified through discussions with ward officials and traditional elders. For FGD with opinion shapers the focus was put on those who were perceived as local experts on livestock matters. To collect qualitative data from animal health professionals, key informant interviews were conducted with Livestock field officers (LFOs), veterinary drug sellers (i.e., agrovet dealers) and Community Animal Health Workers (CAHWs), who provide auxiliary veterinary support services at the community level. CAHWs were conveniently identified when the research team visited villages for interviews. LFOs and agrovet dealers were selected with the assistance of the DVO. See Table 1 for number of qualitative interviews conducted in each category.

**Table 1. The category and number of groups/individuals interviewed in those categories**

| **Category** | **No** |
| --- | --- |
| FGDs | 6 |
| Influential people/Opinion shapers | 10 |
| Agrovets | 5 |
| Livestock Field Officers | 5 |
| CAHWs | 5 |

FGD and KII were concentrated around twelve major themes (see Appendix 1 for specific prompts associated with these themes). The 12 themes related to AMU and AMR and included farm management and economic practices, area disease histories, and knowledge, attitudes, and practices relating to AMU and AMR, including use, governance, regulations, policies, enforcement. The data collection guides were pre-tested and piloted on livestock production systems in Siha District, Kilimanjaro region, on a site resembling proposed Longido pastoral communities. The pilot activities took place between 19 and 21 November 2018 in Siha district (Kilimanjaro region) with similar socioeconomic characteristics with the selected study area**.** Before the pilot, the research team went through the topic guides familiarizing and ensuring that they were translated accordingly. After each field pilot day the research team re-convened and modified the tools accordingly. The pilot was also an opportunity to further improve research qualitative interviewing skills of the recruited research assistants (see further discussion below).

Analysis of the qualitative data was guided by the content thematic analysis approach. In the process of piloting and conducting FGDs/KIIs issues about proposed themes were discussed and documented. This initial analysis allowed for more focused interviewing and discussions in the next sessions. Audio recordings from FGDs and KII were transcribed in to text format. After transcription, word documents (which are a mixture of Kiswahili and Maa) were transcribed to English. After transcription, the data was read and re –read noting down initial and diverse ideas in relation to the themes of discussion and interviews. Initial data analysis was conducted by coding data guided by a deductive framework approach whereby data was coded to the pre-designed categorical themes [14, 15]. From the main themes emerging, sub themes were further coded and collated into potential themes or merged to the main ones. The exercise is currently being repeated extensively as transcripts are transcribed and translated.

Using thematic analysis of qualitative interviews, along with questionnaires from similar studies conducted in the region [16] we developed a KAP survey instrument of over 200 items that included a broad range of demographics, livelihood, health, hygiene and biosecurity topics relating to factors that could promote AMU, sub-optimal AMU practices, and AMR. KAP surveys were administered by a group of four local research assistants using tablets with the Kobo Collect® application. Research assistants fluent in English, Kiswahili and Kimaa were trained in both qualitative and quantitative ethnographic methods. Training occurred over a two-week period. The survey took around 1 hour to complete.

Sampling Procedure

Limitations in time and funding necessitated a purposive selection strategy for quantitative data collection. The sampling design for the KAP survey employed two selection frameworks. First, we targeted adult Maasai men and women at livestock markets within Longido District. This strategy was used given the large distances between Maasai bomas, which constrains the number of interviews that can be conducted. Four livestock markets, Longido, Engarenaibor, Sinya, and Gelailumbwa, representing the major livestock markets in Longido district, were chosen. Enumerators walked around the market and approached adult men and women for interviews. Given the size of these markets (500-1000 people) only a sample of market participants could be surveyed. The strategy of using livestock markets to identify informants was complicated by the length of the survey (1 hr), which dissuaded individuals from participating given they were often preparing themselves to sell or buy items at the market. The second selection framework employed was interviewing Maasai household heads within their bomas. These interviews were planned in advance to ensure Maasai boma heads or spouses were available. Households were targeted using village hamlet leaders passing out information to willing interviewees and also a chain-referral method with referrals being generated by informants and research team members. Within markets and households, enumerators targeted those individuals who were knowledgeable and willing to talk about AMU, purchasing patterns, and livestock management practices. Most ended up being the household heads, ensuring our sample (N=199) was biased towards men.

Ethical Approval

The study was reviewed and approved by the Medical Research Coordinating Committee of the National Institute for Medical Research (NIMR) in Tanzania and certificate clearance no. NIMR/HQ/R.8a/Vol.IX/2926 was issued. Permission to conduct the study in Longido District was sought through sending letters of introductions to respective district authorities whom officially informed selected wards. Following the introduction of the study at the district level via the District Executive Director (DED) with the assistance of the District Veterinary Officer (DVO), the study team formally introduced the project to respective village leaders where the recruitment of study participants was going to take place.

Prior to requesting for consent, an information sheet (IS) containing a detailed narrative of the study and its aims was provided to potential participants who could read, and was read out to those who could not. Participants were informed of the research purposes including the benefits and risks of participation. The respondents were assured of their right to withdraw from study participation at any point, and necessary precautions were made to insure and maintain confidentiality, anonymity and voluntarism throughout the study. A written informed consent was sought from all study participants who could write. For those who could not write a thumbprint signature was requested.

**4. Zambia**

The cross-sectional study was undertaken among 200 broilers farms in five districts (see Figure 4). The five districts are located in two provinces─ Lusaka (Chilanga, Chongwe and Rufunsa) and Central (Chisamba and Chibombo). These districts are divided into farming camps. A total of eight (8) camps from all the districts were selected for the study. Chibombo (Chikumbi and Chibombo East Camps); Chisamba (Chipembi); Chilanga (Mwembeshi and Namalombwe); Chongwe (Chongwe central and Chalimbana) and Rufunsa (Nangwena). The districts in Zambia were visited between March 1^st^ and March 15^th^ 2019. These locations were selected given high numbers of broiler farmers.

Figure 4. Map of study area in Zambia. Maps were created using ArcGIS software by Esri. The base map is sourced from Esri and modified in ArGIS Pro. "Light Gray Canvas" [basemap] https://www.arcgis.com/home/item.html?id=ee8678f599f64ec0a8ffbfd5c429c896.. The administrative later was sourced from MapLibrary http://www.maplibrary.org/library/stacks/Africa/index.htmFeb 19th, 2019.


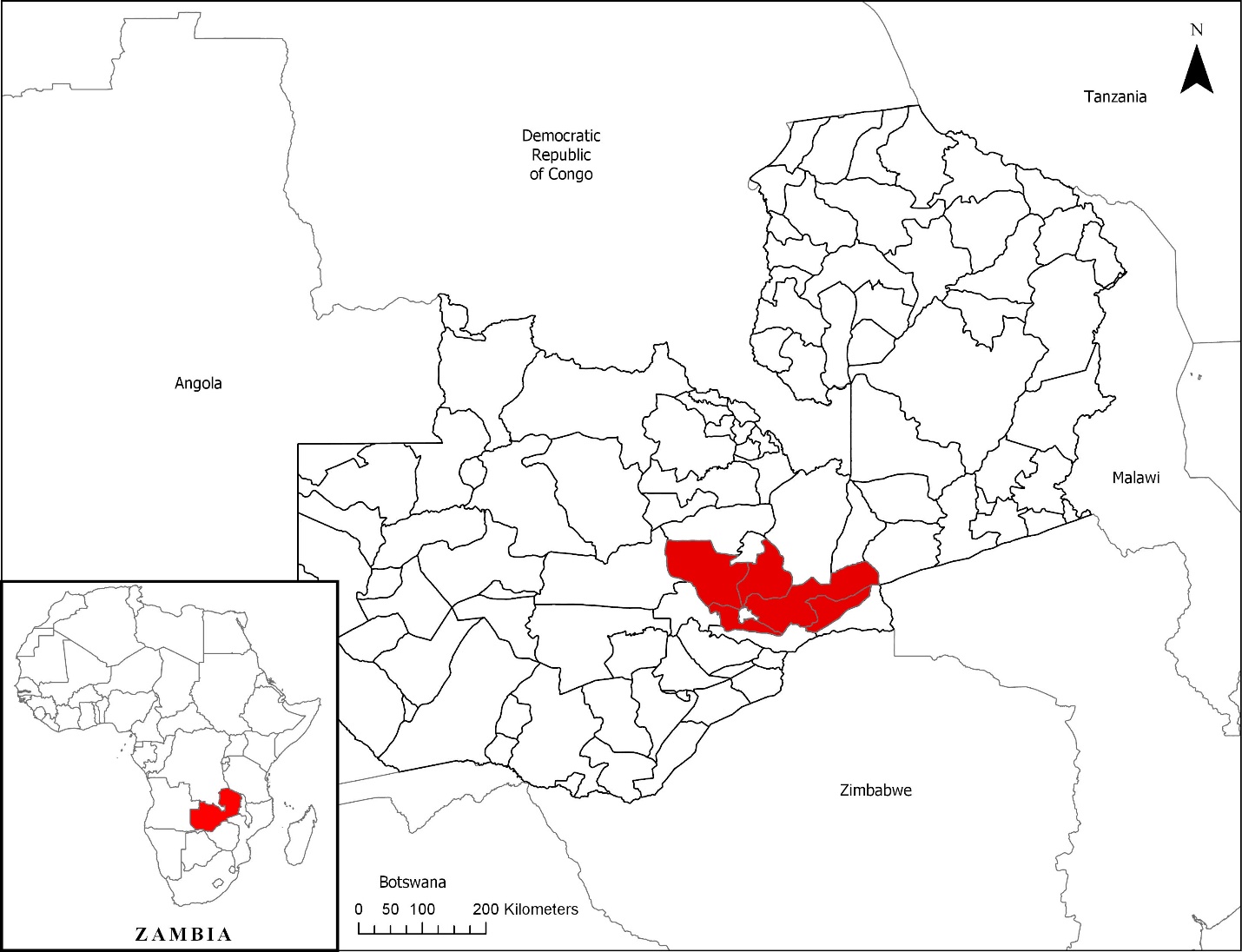


## KAP Survey Development

An interdisciplinary research team comprised of animal health experts and social scientists from the FAO, the Ministry of Health, Ministry of Fisheries and Livestock (Department of Veterinary Services), the University of Zambia, The Zambia Medicines Regulatory Authority (ZAMRA) and the Zambia Community Health Initiative used a mixed-methods approach to develop the KAP survey. The study employed a slightly modified version of the exploratory cross-sectional survey design approach [13] using both qualitative and qualitative data to inform quantitative parameters and confirm quantitative measures. Qualitative data collection included six FGDs with broiler farmers who were mostly heads of households and farm workers, four district veterinary officers/one district livestock officer, and five veterinary assistants. FGD and KIIs were concentrated around twelve major themes relating to AMU and AMR including farm management and economic practices, disease histories, and knowledge, attitudes, and practices relating to AMU and AMR, including use, governance, regulations, policies, and enforcement. Thematic analysis of qualitative interviews were used to developed a KAP survey instrument of over 200 items that included a broad range of demographics, livelihood, health, hygiene and biosecurity topics relating to factors that could promote AMU, sub-optimal AMU practices, and AMR. KAP questionnaires were administered by a group of eight local research assistants using tablets with the Kobo Collect® application. These assistants were veterinary assistants working in the study districts. While the veterinary assistants worked in the districts, they conducted surveys in areas where they had not worked before to limit bias. Enumerators participated in a four-day training and KAP piloting in Kabwe, Zambia.

**Sampling Procedure**

The sampling frame was generated by enumerators developing lists of all broiler farmers living in the eight selected camps. From these lists, we randomly sampled 25 households per camp to reach the 200 household desired sample size (see Table 2 for numbers of surveyed households per camp). The KAP Interviews lasted around 1 hour. All informants provided consent to participate through signature or thumbprint. The survey was primarily administered in local languages (Bemba and Nyanja) and in some cases, code-switching between local languages and English.

Table 2. Surveyed Camps

| **Camp** | **Frequency** |
| --- | --- |
| Chukumbi | 25 |
| Chibombo East Camp | 25 |
| Chipembi | 26 |
| Namalombwe | 23 |
| Chongwe Central | 24 |
| Chalimbawa | 26 |
| Mwembeshi | 25 |
| Ngawena | 25 |
| **Total:** | **199** |

## Ethical Approval

The study was reviewed and approved by the ERES Converge Ethic Committee,Ref: No 2018-Nov -020 was issued. Prior to requesting for consent, an information sheet (IS) containing a detailed narrative of the study and its aims was provided to potential participants who could read, and was read out to those who could not. Participants were informed of the research purposes including the benefits and risks of participation. The respondents were assured of their right to withdraw from study participation at any point, and necessary precautions were made to insure and maintain confidentiality, anonymity and voluntarism throughout the study. A written informed consent was sought from all study participants who could write. For those who could not write a thumbprint signature was requested.

**5. Zimbabwe**

This study was carried out in six districts in northern Zimbabwe namely Mutare, Mutasa, Marondera, Murehwa, Zvimba and Chegutu Districts (see Figure 5). Significant segments of the population within these districts engage in medium to small-scale broiler production.

Figure 5. Map of study area. Sampled districts are shaded in red. Maps were created using ArcGIS software by Esri. The base map is sourced from Esri and modified in ArGIS Pro. "Light Gray Canvas" [basemap] https://www.arcgis.com/home/item.html?id=ee8678f599f64ec0a8ffbfd5c429c896. The administrative later was sourced from MapLibrary <http://www.maplibrary.org/library/stacks/Africa/index.htm> Nov 10th, 2019.


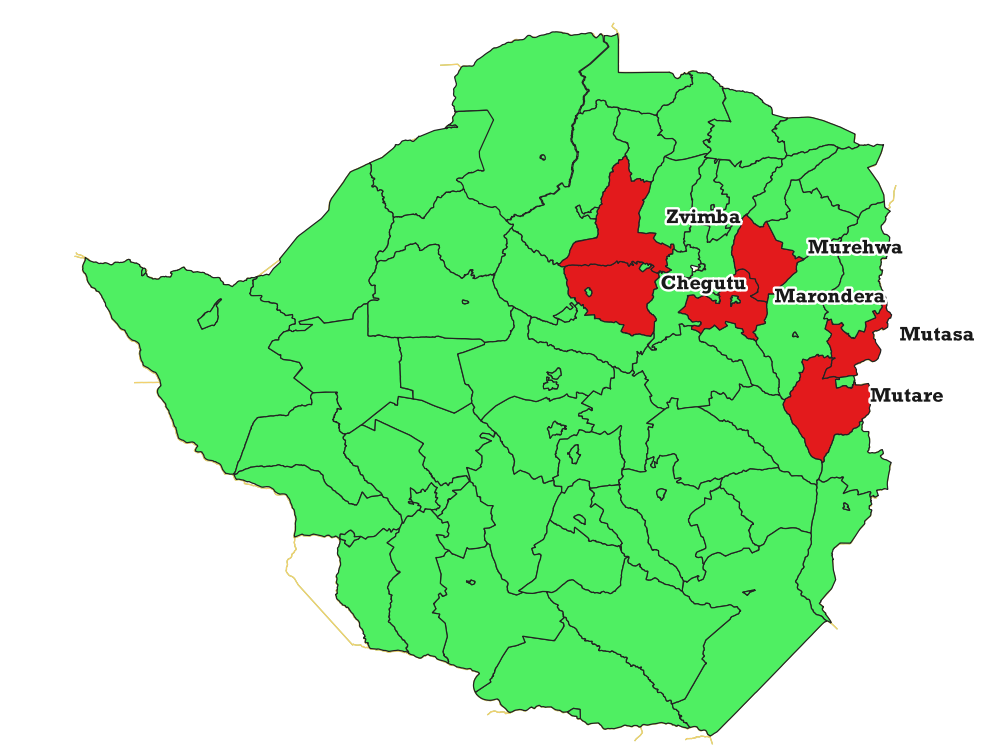


**Mutasa district**

Mutasa is located 30 km north of Mutare and stretches up to the Honde Valley, which is about 100 km north-east of Mutare. Mutasa has a total human population of 169,000 with the majority being farmers as the district’s economy is agro-based. The district lies in Natural Region 2B (Agro-ecological classification) and experiences high temperatures (above 25 degrees Celsius) and rainfall that ranges between 650-800mm per year.  Villages raise cattle, goats and chicken; and they grow mostly maize, groundnuts and sugarcane and have plantations for fruit trees in the fields. Some of the villages are small landholder growers of coffee, tea and banana plantations. Large scale commercial plantations produce timber, coffee and tea.

**Mutare district**

Mutare district lies in the eastern part of the country. It has a total human population of 448,810 and lies in Natural Region 1 - 5. The Burma Valley and Vumba areas lie in Natural Region 1 and receives high rainfall (more than 1000mm per annum); the Zimunya and Odzi areas lie in Natural Region 3, receiving an average rainfall of 650 – 800mm per annum; the Marange area lies in Natural Region 5 and receives very low rainfall (below 600mm per annum).

**Marondera district**

Marondera district lies in the eastern part of the country. It has a total human population of 199,607 with the majority in the district’s rural areas being farmers. The district lies in Natural Region 2B. This sub-region receives an average of 16-18 rainy pentads per season and is subject either to rather more severe dry spells during the rainy season or to the occurrence of relatively short rainy seasons. In either event, crop yields in certain years will be affected, but not sufficiently and frequently to change the overall utilization from intensive systems of farming.

**Murehwa district**

Murehwa district which lies North-eastern part of the country, has a total human population of 165,788 with a large part of the district being communal areas. The district lies in Natural Region 3 and experiences high temperatures (above 25 degrees Celsius). Rainfall in this region is moderate in total amount (650-800mm), but, because much of it is accounted for by infrequent heavy falls and temperatures are generally high, its effectiveness is reduced. This region will receive an average of 14-16 rainy pentads per season. The region is also subject fairly severe mid-season dry spells and therefore is marginal for maize, tobacco and cotton production, or for enterprises based on crop production alone. The farming systems, in conformity with the natural conditioning factors, should therefore be based on both livestock production (assisted by the production of fodder crops) and cash crops under good management on soils of high available moisture potential.

**Zvimba district**

Zvimba district lies in the central northern part of the country. The district has a total human population of 245,489. The district lies in Natural Region 2A (Agro-ecological classification) with rainfall pattern ranging between 750mm to 1000mm. The region is suitable for intensive systems of farming based on crops and /or livestock production.

**Chegutu district**

Chegutu district lies in the central northern part of the country. The district has a total human population of 224,589. It lies in Natural Region 2B (Agro-ecological classification) and experiences high temperatures (above 25 degrees Celsius) and rainfall that ranges between 650-800mm per year.

**KAP Study Development**

Development of the KAP survey proceeded through thematic analysis of focus group discussions (FGDs) and key informant interviews (KIIs). FGD’s and KII’s participants were selected purposively based upon recommendations from local government officials and community leaders. Eight key informant (KIIs) interviews were conducted with the following; two feed manufacturing companies, two branches of a veterinary medicines wholesale and retail company, one broiler contract grower managing company, two broiler farmers contracted under the Command Poultry Programme and officials from the Departments of Crop and Livestock & Veterinary Services in the selected districts. The final number of KIIs was determined by the variability of answers attained during sessions.

FGDs were conducted with broiler farmers of mixed gender, ensuring a balanced participation from both sexes. A total of three focus group discussions were done, with the final number also determined by the variability of information received from the FGDs. All FGDs and KIIs were conducted by a social science expert with extensive experience in conducting qualitative interviews in order to moderate the discussions and also ensure participants felt comfortable during sessions. FGDs included a second researcher who recorded responses upon receipt of consent.

Thematic analysis of FGDs and KIIs was combined with results of a literature review to develop the KAP tool (questionnaire). The questionnaire covered the following areas; farmer demographics, general husbandry practices, anti-microbial usage, biosecurity and other poultry disease management strategies. The questionnaire was translated into Shona and then back-translated by a two member-team fluent in English and Shona to ensure correct translation. The questionnaire was pre-tested on ten farmers based in Harare to ensure question clarity and to estimate the time demands of the survey.

The questionnaire consisted of closed and open ended questions and was initially designed using XLS form coding on a Microsoft Excel spreadsheet and then uploaded into Samsung tablets using Kobo Collect® application. A total of twelve enumerators, two per district, were selected to conduct the data collection process. Enumerators were drawn from the Departments of Crop and Livestock & Veterinary Services. The selected numerators were trained in ethnographic methods (e.g., proper survey etiquette) and on mobile survey data collection processes across a one-week period at the FAO’s sub-regional office in Harare. During training, each enumerator conducted test interviews with the same people (N=3 interviews total) and responses were assessed using Cronbach’s Alpha to ensure survey comprehension across enumerators. Enumerators administered the questionnaires using tablets, in English or Shona vernacular depending on the farmer’s preference.

Following survey development and enumerator training, the selected farmers in Mutare, Mutasa, Marondera, Murehwa, Zvimba and Chegutu districts were interviewed at their households. Prior to survey administration, each farmer was provided with an introduction to the project and told that all information would remain confidential. They were also told that it was not mandatory to participate, and that they could end the interview at any time (see 3.5 Ethical Consideration and Recruitment Procedures for further information). After providing written consent (see attached Informed Consent Form) the survey was administered, and took an average of 1hour per session. During the interviews, farmers were asked to show any medicines they were using, or empty containers of previously used medicines, if they were available. The medicine containers were photographed.

**Sampling procedure**

The initial sampling frame was obtained from census lists of broiler farmers provided by the Ministry of Lands, Agriculture, Climate, Water and Rural Resettlement and Zimbabwe National Statistics Agency. Consultation of the list alongside discussions with local animal health professionals, however, indicated that many names on the list were no longer participating in broiler production. As such, names identified as keeping broilers were approached and asked for names of individuals who were still active in broiler production. This technique was employed until a total of fifty farmers were selected for interview per district, for the six districts, giving a total of three hundred farmers.

**Ethical approval**

Ethical clearance and permission to conduct the study in the Mutare, Mutasa, Marondera, Murehwa, Zvimba and Chegutu districts was obtained from the Agriculture Research Council (ARC) of the Department of Veterinary Services & Crop and Livestock (Reference Number: 008/2018). Prior to requesting for consent, an information sheet (IS) containing a detailed narrative of the study and its aims was provided to potential participants who could read, and was read out to those who could not. Participants were informed of the research purposes including the benefits and risks of participation. The respondents were assured of their right to withdraw from study participation at any point, and necessary precautions were made to insure and maintain confidentiality, anonymity and voluntarism throughout the study. A written informed consent was sought from all study participants who could write. For those who could not write a thumbprint signature was requested.

Table 3. **Associations between KAP measures and demographics, on-farm dynamics, health seeking practices and country controls in poultry systems.** Results were Bootstrapped 200 times using the vce option in Stata 16.1.

| VARIABLES | Knowledge | Attitudes | Practices |
| --- | --- | --- | --- |
| **Agrovet_advice** |  |  |  |
| - Sometimes | 0.038* | 0.004 | 0.007 |
| **Agrovet_advice** | (-0.006 - 0.083) | (-0.030 - 0.038) | (-0.011 - 0.024) |
| - Almost always | 0.005 | 0.012 | -0.016+ |
| **Extension advice** | (-0.052 - 0.062) | (-0.031 - 0.056) | (-0.040 - 0.008) |
| - Sometimes | -0.001 | 0.010 | -0.022* |
| **Extension advice** | (-0.060 - 0.057) | (-0.027 - 0.048) | (-0.048 - 0.004) |
| - Almost always | 0.051+ | 0.029 | -0.050*** |
| **Private_vet advice** | (-0.012 - 0.115) | (-0.020 - 0.078) | (-0.078 - -0.022) |
| - Sometimes | 0.022 | -0.037 | 0.003 |
| **Private_vet_advice** | (-0.078 - 0.123) | (-0.100 - 0.027) | (-0.036 - 0.042) |
| - Almost Always | 0.024 | -0.027 | 0.032** |
| **Laboratory_advice** | (-0.052 - 0.100) | (-0.087 - 0.034) | (0.001 - 0.062) |
| - Sometimes | 0.042 | -0.039 | 0.001 |
| **Laboratory_advice** | (-0.037 - 0.121) | (-0.110 - 0.032) | (-0.053 - 0.055) |
| -Almost always | -0.167+ | -0.013 | -0.039 |
| **Government vet_advice** | (-0.373 - 0.038) | (-0.171 - 0.145) | (-0.123 - 0.046) |
| - Sometimes | 0.088*** | 0.041** | 0.001 |
| **Government vet_advice** | (0.038 - 0.139) | (0.004 - 0.078) | (-0.021 - 0.023) |
| - Almost always | 0.135*** | 0.063*** | 0.066*** |
| **Friends_advice** | (0.084 - 0.185) | (0.017 - 0.109) | (0.042 - 0.090) |
| -Sometimes | 0.002 | 0.004 | -0.007 |
| **Friends_advice** | (-0.044 - 0.047) | (-0.030 - 0.039) | (-0.025 - 0.011) |
| -Almost always | 0.004 | -0.010 | -0.000 |
|  | (-0.044 - 0.052) | (-0.044 - 0.024) | (-0.022 - 0.021) |
| Farm scale (std) | 0.026+ | -0.000 | -0.000 |
|  | (-0.010 - 0.062) | (-0.032 - 0.031) | (-0.014 - 0.013) |
| AMs used per month | -0.004 | -0.017+ | 0.005 |
|  | (-0.031 - 0.023) | (-0.039 - 0.005) | (-0.005 - 0.016) |
| Treatment failure | 0.046** | -0.012 | -0.012+ |
|  | (0.008 - 0.085) | (-0.045 - 0.021) | (-0.029 - 0.005) |
| Disease level | -0.030 | 0.040 | -0.049+ |
|  | (-0.189 - 0.128) | (-0.073 - 0.154) | (-0.119 - 0.020) |
| Number of AM medicines | 0.001+ | 0.000 | 0.000* |
|  | (-0.000 - 0.002) | (-0.001 - 0.001) | (-0.000 - 0.001) |
| Keep records | 0.084*** | 0.012 | 0.013 |
|  | (0.033 - 0.134) | (-0.028 - 0.052) | (-0.008 - 0.034) |
| Training | 0.162*** | 0.032** | -0.030*** |
|  | (0.118 - 0.207) | (0.002 - 0.063) | (-0.049 - -0.012) |
| Keeping time (yrs) | 0.004*** | 0.000 | 0.001+ |
|  | (0.001 - 0.006) | (-0.002 - 0.002) | (-0.000 - 0.002) |
| Gender (1=Female) | -0.042* | -0.001 | 0.009 |
|  | (-0.085 - 0.001) | (-0.032 - 0.031) | (-0.008 - 0.026) |
| Age | 0.001 | 0.001** | -0.000 |
|  | (-0.001 - 0.002) | (0.000 - 0.002) | (-0.001 - 0.001) |
| Primary Education | -0.051 | 0.005 | -0.001 |
|  | (-0.151 - 0.049) | (-0.090 - 0.100) | (-0.061 - 0.059) |
| Secondary Education | -0.011 | 0.059+ | 0.017 |
|  | (-0.101 - 0.079) | (-0.028 - 0.146) | (-0.042 - 0.076) |
| Tertiary Education | 0.014 | 0.070+ | 0.022 |
|  | (-0.088 - 0.115) | (-0.021 - 0.160) | (-0.037 - 0.081) |
| Kenya | 0.162*** | 0.053+ | 0.118*** |
|  | (0.061 - 0.262) | (-0.024 - 0.130) | (0.071 - 0.166) |
| Zambia | 0.104** | 0.058+ | 0.165*** |
|  | (0.016 - 0.192) | (-0.022 - 0.138) | (0.125 - 0.206) |
| Zimbabwe | 0.013 | -0.010 | 0.180*** |
|  | (-0.056 - 0.083) | (-0.067 - 0.048) | (0.145 - 0.215) |
| Constant | 0.223*** | 0.523*** | 0.649*** |
|  | (0.086 - 0.360) | (0.389 - 0.657) | (0.575 - 0.723) |
|  |  |  |  |
| Observations | 665 | 665 | 665 |
| R-squared | 0.266 | 0.084 | 0.358 |

ci in parentheses

*** p<0.01, ** p<0.05, * p<0.10, + p<0.20

**Table 4.** **Associations between KAP measures and demographics, on-farm dynamics, health seeking practices in pastoralist systems.** Results were Bootstrapped 200 times using the vce option in Stata 16.1.

| VARIABLES | Knowledge | Attitudes | Practices |
| --- | --- | --- | --- |
| **Agrovet_advice** |  |  |  |
| - Sometimes | 0.086* | -0.013 | 0.004 |
| **Agrovet_advice** | (-0.007 - 0.180) | (-0.066 - 0.041) | (-0.053 - 0.062) |
| - Almost always | 0.022 | -0.018 | 0.026 |
| **Extension advice** | (-0.148 - 0.193) | (-0.114 - 0.078) | (-0.043 - 0.094) |
| - Sometimes | -0.025 | 0.058** | -0.009 |
| **Extension advice** | (-0.107 - 0.057) | (0.002 - 0.114) | (-0.054 - 0.036) |
| - Almost always | 0.004 | -0.019 | -0.013 |
| **Laboratory_advice** | (-0.294 - 0.303) | (-0.192 - 0.154) | (-0.131 - 0.104) |
| - Sometimes | -0.105 | 0.091+ | 0.007 |
| **Laboratory_advice** | (-0.342 - 0.131) | (-0.019 - 0.200) | (-0.111 - 0.124) |
| -Almost always | -0.118* | -0.090** | -0.195*** |
| **Government vet_advice** | (-0.242 - 0.007) | (-0.158 - -0.021) | (-0.265 - -0.126) |
| - Sometimes | 0.009 | 0.002 | -0.020 |
| **Government vet_advice** | (-0.077 - 0.095) | (-0.056 - 0.060) | (-0.067 - 0.028) |
| - Almost always | 0.174* | 0.005 | -0.047 |
| **Friends_advice** | (-0.004 - 0.352) | (-0.087 - 0.096) | (-0.146 - 0.053) |
| -Sometimes | 0.038 | -0.067 | -0.060 |
| **Friends_advice** | (-0.131 - 0.207) | (-0.198 - 0.064) | (-0.179 - 0.060) |
| -Almost always | 0.026 | -0.025 | -0.027 |
|  | (-0.146 - 0.197) | (-0.154 - 0.104) | (-0.153 - 0.098) |
| Farm scale (std) | -0.020 | -0.009 | 0.018+ |
|  | (-0.065 - 0.026) | (-0.042 - 0.025) | (-0.009 - 0.045) |
| AMs used per month | 0.007 | -0.009 | -0.049*** |
|  | (-0.038 - 0.053) | (-0.049 - 0.031) | (-0.083 - -0.016) |
| Treatment failure | 0.017 | 0.002 | -0.064*** |
|  | (-0.067 - 0.101) | (-0.054 - 0.058) | (-0.110 - -0.019) |
| Disease level | 0.104 | 0.306*** | -0.433*** |
|  | (-0.207 - 0.415) | (0.094 - 0.518) | (-0.639 - -0.228) |
| Number of AM medicines | 0.001 | -0.000 | -0.003 |
|  | (-0.019 - 0.021) | (-0.014 - 0.013) | (-0.013 - 0.008) |
| Keep records | 0.039 | 0.020 | 0.010 |
|  | (-0.054 - 0.133) | (-0.042 - 0.083) | (-0.046 - 0.065) |
| Training | -0.041 | -0.083 | -0.052 |
|  | (-0.167 - 0.084) | (-0.234 - 0.069) | (-0.190 - 0.086) |
| Gender (1=Female) | 0.053 | 0.048 | -0.024 |
|  | (-0.109 - 0.215) | (-0.057 - 0.154) | (-0.092 - 0.043) |
| Age | -0.006*** | -0.001 | 0.001 |
|  | (-0.009 - -0.003) | (-0.003 - 0.001) | (-0.001 - 0.003) |
| Primary Education | 0.054+ | -0.005 | -0.033 |
|  | (-0.027 - 0.135) | (-0.055 - 0.046) | (-0.090 - 0.024) |
| Secondary Education | 0.041 | 0.080** | 0.021 |
|  | (-0.113 - 0.196) | (0.004 - 0.157) | (-0.068 - 0.111) |
| Tertiary Education | 0.248*** | 0.008 | 0.014 |
|  | (0.114 - 0.381) | (-0.321 - 0.338) | (-0.127 - 0.154) |
| Constant | 0.615*** | 0.441*** | 0.818*** |
|  | (0.387 - 0.843) | (0.269 - 0.613) | (0.701 - 0.934) |
|  |  |  |  |
| Observations | 194 | 194 | 194 |
| R-squared | 0.216 | 0.196 | 0.385 |

**Table 5. Demographic and Socioeconomics of Study Sample.** Tertiary and above indicates any additional education after secondary school, including certificates, diplomas, bachelors, masters, and PhDs. Keeping time is the number of years that a farmer has been engaged in the targeted production system. **Bold text** indicates variable was included in regression models.

|  | **Ghana** | | **Zimbabwe** | | **Kenya** | | **Zambia** | | **Tanzania** | |
| --- | --- | --- | --- | --- | --- | --- | --- | --- | --- | --- |
|  | Layer | | Broiler | | Layer | | Broiler | | Cattle/Shoats | |
|  | mean | sd | mean | sd | mean | sd | mean | sd | mean | sd |
| **Age** | 36.67 | 13.02 | 48.23 | 12.47 | 49.34 | 14.95 | 45.18 | 13.77 | 47.68 | 13.72 |
| **Gender (1=Female, 0=Male)** | 0.23 | 0.42 | 0.25 | 0.43 | 0.53 | 0.50 | 0.39 | 0.49 | 0.07 | 0.25 |
| Household Size | 5.28 | 2.89 | 5.39 | 1.66 | 4.80 | 2.98 | 5.41 | 3.49 | 8.55 | 4.33 |
| Read (1=Yes, 0=No) | 0.99 | 0.10 | 0.99 | 0.08 | 1.00 | 0.00 | 0.96 | 0.19 | 0.78 | 0.41 |
| ***Education Level*** | % | | % | | % | | % | | % | |
| - None | 10.00 | | 1.04 | | 0.00 | | 0.00 | | 61.54 | |
| - Primary (1-5 years) | 40.00 | | 10.38 | | 35.53 | | 25.66 | | 29.74 | |
| - Secondary (6-12 years) | 31.82 | | 69.44 | | 38.16 | | 44.44 | | 6.67 | |
| - Tertiary and above* | 18.18 | | 19.10 | | 26.32 | | 18.12 | | 2.05 | |
| *Respondent Position* | % | | % | | % | | % | | % | |
| - Farm Owner | 40.91 | | 47.75 | | 59.21 | | 74.24 | | 85.13 | |
| - Farm Manager | 59.09 | | 0.00 | | 7.89 | | 0.00 | | 0.00 | |
| - Spouse of Owner | 0.00 | | 34.26 | | 28.95 | | 19.70 | | 13.85 | |
| - Son/Daughter | 0.00 | | 17.99 | | 3.95 | | 6.06 | | 1.03 | |
|  | mean | sd | mean | sd | mean | sd | mean | sd | mean | sd |
| Broilers | 130.58 | 958.23 | 278.27 | 1071.14 | 5.54 | 16.00 | 738.36 | 6768.10 | 0.00 | 0.00 |
| Layers | 9037.43 | 14795.41 | 12.91 | 75.88 | 1079.59 | 2292.61 | 36.86 | 224.72 | 0.00 | 0.00 |
| Local Chickens | 1.55 | 7.59 | 25.10 | 71.72 | 15.24 | 32.81 | 26.57 | 101.01 | 3.37 | 12.80 |
| Other Fowl (geese, duck, turkey) | 3.09 | 26.14 | 1.44 | 4.64 | 0.14 | 0.86 | 7.23 | 34.70 | 0.00 | 0.00 |
| Cattle (Dairy and Beef) | 2.73 | 12.85 | 4.78 | 15.13 | 3.18 | 3.17 | 3.08 | 9.66 | 147.43 | 241.82 |
| Shoats (sheep and goats) | 10.34 | 29.17 | 3.28 | 9.52 | 1.49 | 2.18 | 6.73 | 15.60 | 233.16 | 453.86 |
| Pigs | 3.27 | 9.80 | 0.73 | 4.90 | 0.91 | 3.46 | 10.20 | 36.24 | 0.00 | 0.00 |
| Donkeys | 0.00 | 0.00 | 0.04 | 0.60 | 0.00 | 0.00 | 0.04 | 0.39 | 3.76 | 6.62 |
| Dogs | 1.35 | 2.32 | 1.04 | 1.57 | 1.13 | 1.21 | 1.26 | 2.18 | 2.34 | 3.14 |
| N | 110 | | 289 | | 76 | | 198 | | 195 | |

**Table 6. Management Practices for poultry systems. Bold text** indicates variable was included in regression models.

|  | **Ghana** | | **Zimbabwe** | | **Kenya** | | **Zambia** | |
| --- | --- | --- | --- | --- | --- | --- | --- | --- |
|  | mean | sd | mean | sd | mean | sd | mean | sd |
| Mixed age cohorts (1=Yes, 0=No) | 0.44 | 0.50 | 0.70 | 0.46 | 0.36 | 0.48 | 0.88 | 0.33 |
| How long have you kept layers/broilers (years) | 9.35 | 7.30 | 6.86 | 8.82 | 7.41 | 7.74 | 10.88 | 8.39 |
| Average mortality | 14 | 0.16 | 8.47 | 12.21 | 16.40 | 17.14 | 10.26 | 0.97 |
| Layer house number ((1=Yes, 0=No) | 11.34 | 15.78 | 1.74 | 1.45 | 1.73 | 1.44 | 2.39 | 1.68 |
| Layer house has two-open sides (1=Yes, 0=No) | 0.73 | 0.45 | 0.53 | 0.50 | 0.48 | 0.50 | 0.97 | 0.16 |
| Layer house has 50 cm wall (1=Yes, 0=No) | 0.93 | 0.26 | 0.66 | 0.48 | 0.33 | 0.47 | 0.92 | 0.27 |
| Layer house is 10 meters from other structures | 0.62 | 0.49 | 0.31 | 0.46 | 0.22 | 0.41 | 0.00 | 0.00 |
| Footbath present at poultry house (1=Yes, 0=No) | 0.06 | 0.25 | 0.22 | 0.41 | 0.03 | 0.17 | 0.48 | 0.50 |
| Farm has boots (1=Yes, 0=No) | 0.66 | 0.47 | 0.32 | 0.47 | 0.72 | 0.45 | 0.77 | 0.42 |
| Farm has overalls (1=Yes, 0=No) | 0.28 | 0.45 | 0.28 | 0.45 | 0.37 | 0.49 | 0.49 | 0.51 |
| Medicine costs per bird per cycle | 0.65 | 1.61 | 0.31 | 0.49 | 0.21 | 0.18 | 0.23 | 0.43 |
| **Keep farm records** | 0.99 | 0.01 | 0.71 | 0.45 | 0.59 | 0.50 | 0.78 | 0.42 |
| **Training** | 0.66 | 0.47 | 0.65 | 0.48 | 0.36 | 0.48 | 0.42 | 0.50 |
| **Treatment failure** | 0.60 | 0.49 | 0.72 | 0.45 | 0.61 | 0.48 | 0.22 | 0.42 |
| **AMU** | 1.04 | 0.47 | 0.41 | 0.57 | 0.41 | 0.57 | 2.27 | 1.04 |
| **Number of AM Medicines** | 9.05 | 5.05 | 2.35 | 2.58 | 5.30 | 3.53 | 3.77 | 2.96 |
| N | 110 |  | 289 |  | 76 |  | 198 |  |

**Table 7. Management Practices for cattle, sheep, and goats (shoats).** Money spent is calculated per head and is presented in USD.

|  | mean | sd | min | max |
| --- | --- | --- | --- | --- |
| Cattle moving in and out of boma | 65.21 | 101.84 | 0.00 | 800.00 |
| Cattle staying in boma | 38.31 | 86.64 | 0.00 | 560.00 |
| Cattle in temporary bomas | 64.41 | 120.34 | 0.00 | 1000.00 |
| Cattle Sold last season | 12.03 | 22.76 | 0.00 | 200.00 |
| Cattle bought last season | 1.68 | 3.32 | 0.00 | 22.00 |
| Shoats moving in and out of boma | 131.36 | 230.38 | 1.00 | 2700.00 |
| Shoats staying in the boma | 84.21 | 225.25 | 0.00 | 2700.00 |
| Shoats in temporary boma | 17.59 | 84.56 | 0.00 | 1000.00 |
| Shoats bought last season | 8.46 | 13.46 | 0.00 | 80.00 |
| Shoats sold last season | 26.20 | 50.08 | 0.00 | 600.00 |
| Money spent on medicine per head: Wet season | 1.26 | 2.38 | 0.00 | 25.00 |
| Money spent on medicine per head: Dry season | 1.20 | 2.28 | 0.00 | 25.00 |
| **Training** | 0.04 | 0.05 | 0.00 | 1.00 |
| **Keep Farm Records (1=Yes 0=No)** | 0.10 | 0.31 | 0.00 | 1.00 |
| **AMU** | 1.66 | 0.78 | 1.00 | 4.00 |
| **Num of AMU medicines** | 4.51 | 2.18 | 1.00 | 11.00 |
| N | 195 |  |  |  |

**Table 8. AM use by country.** Antimicrobial use was used in models as **“AMU”**

| **Country** | **AM use per month** | **Frequency** | **Percentage (%)** |
| --- | --- | --- | --- |
| Ghana N=110 | Never | 10 | 9.09 |
|  | One or Two | 85 | 77.27 |
|  | 3 to 5 times | 15 | 13.64 |
| Kenya N=76 | Never | 48 | 63.16 |
|  | One or Two | 25 | 32.89 |
|  | 3 to 5 times | 3 | 3.95 |
| Tanzania N=195 | One or Two | 96 | 49.23 |
|  | 3 to 5 times | 76 | 38.97 |
|  | 6 to 10 times | 16 | 8.21 |
|  | Over 10 times | 7 | 3.59 |
| Zambia N=196 | One or Two | 48 | 24.49 |
|  | 3 to 5 times | 84 | 42.86 |
|  | 6 to 10 times | 26 | 13.27 |
|  | Over 10 times | 38 | 19.39 |
| Zimbabwe N=288 | Never | 36 | 12.50 |
|  | One or Two | 214 | 74.31 |
|  | 3 to 5 times | 35 | 12.15 |
|  | 6 to 10 times | 3 | 1.04 |

**Table 9. Most commonly reported diseases in layers, broilers, cattle and sheep and goats.** Maasai disease names are often syndromic and for these we provide a translation into English. Descriptions of diseases can be found in MSD veterinary manual <https://www.msdvetmanual.com/>.

|  | **Layers** | | **Broilers** | |  | **Cattle** | | **Sheep and Goats** | |
| --- | --- | --- | --- | --- | --- | --- | --- | --- | --- |
| Diseases | % of  HH | N | % of  HH | N | Diseases | % of  HH | N | % of  HH | N |
|  |  |  |  |  |  |  |  |  |  |
| Coccidiosis | 63 | 117 | 43 | 209 | Contagious Bovine/Caprine Pleuropneumonia | 70 | 137 | 92 | 179 |
| Coryza | 18 | 33 | 03 | 15 | Coenurosis | 63 | 123 | 96 | 187 |
| Chronic Respiratory Diseases | 76 | 141 | 32 | 156 | East Coast Fever | 61 | 119 | 03 | 6 |
| Fowl Pox | 14 | 26 | 02 | 10 | Foot and Mouth Disease | 59 | 115 | 29 | 57 |
| Fowl Typhoid | 6 | 11 | 00 | 0 | Sheep and Goat Pox | 55 | 107 | 54 | 105 |
| Gumboro | 32 | 60 | 12 | 58 | Nunuk Three-day sickness | 42 | 82 | 1 | 2 |
| Infectious Bronchitis | 05 | 9 | 30 | 146 | Olarashiskashi | 41 | 80 | 4 | 8 |
| Marek’s Disease | 05 | 9 | 0 | 0 | Olkirikiri- Three day sickness | 39 | 76 | 13 | 25 |
| Newcastle | 39 | 73 | 20 | 97 | Embongiti | 32 | 62 | 27 | 53 |
| Ectoparasites | 0 |  | 5 | 24 | Anthrax | 29 | 57 | 28 | 55 |
| Birds never get disease | 2 | 4 | 8 | 39 | Orkutro | 26 | 51 | 33 | 64 |
| Don’t know any diseases | 5 | 9 | 10 | 49 | Lipis_Nagana_Ndorobo | 24 | 47 | 10 | 20 |
| N | 186 |  | 487 |  | Babebiosis | 24 | 47 | 6 | 12 |
|  |  |  |  |  | Olodwaa_Olomorroj | 21 | 41 | 2 | 4 |
|  |  |  |  |  | Malignant Catarrhal Fever | 12 | 23 | 0 | 0 |
|  |  |  |  |  | Emonua | 8 | 16 | 0 | 0 |
|  |  |  |  |  | Olmok | 8 | 16 | 1 | 2 |
|  |  |  |  |  | Black Quarter | 7 | 14 | 0 | 0 |
|  |  |  |  |  | Peste des Petit Ruminants | 6 | 12 | 0 | 0 |
|  |  |  |  |  | Lumpy Skin Disease | 4 | 8 | 2 | 4 |
|  |  |  |  |  | Brucolis | 2 | 4 | 1 | 2 |
|  |  |  |  |  | Animals never get disease | 0 | 0 | 0 | 0 |
|  |  |  |  |  | Don’t know any diseases | 0 | 0 | 0 | 0 |
|  |  |  |  |  | N | 195 |  | 195 |  |

**Table 10. Where antimicrobials are usually acquired.** Pooled across countries.

| Source | % of HH reporting | N |
| --- | --- | --- |
| Agrovet | 83 | 720 |
| Feed Distributor | 13 | 113 |
| Shop that is not an agrovet | 12 | 104 |
| Government Veterinarian | 11 | 95 |
| Friends | 8 | 69 |
| Private veterinarian | 6 | 52 |
| Market | 6 | 52 |
| Community Health Worker | 1 | 9 |
| Middleman | 1 | 9 |
| Never purchased | 1 | 9 |
| N |  | 887 |

**Table 11. Sources of antimicrobials by country.** Advice was a categorical value with 0=none/rarely, 1=sometimes, 2=almost always.

|  | **Ghana** | | **Kenya** | | **Tanzania** | | **Zambia** | | **Zimbabwe** | | **Pooled** |
| --- | --- | --- | --- | --- | --- | --- | --- | --- | --- | --- | --- |
|  | mean | sd | mean | sd | mean | sd | mean | sd | mean | sd |  |
| Extension_worker | 0.00 | 0.00 | . | . | 0.02 | 0.14 | . | . | 0.01 | 0.10 |  |
| Agrovet | 0.95 | 0.23 | 0.96 | 0.20 | 0.83 | 0.38 | 0.86 | 0.35 | 0.74 | 0.44 |  |
| Government_vet | 0.01 | 0.10 | 0.03 | 0.16 | 0.23 | 0.42 | . | . | 0.09 | 0.28 |  |
| Private_vet | 0.01 | 0.10 | 0.05 | 0.22 | 0.20 | 0.40 | 0.01 | 0.10 | 0.01 | 0.12 |  |
| Friend | 0.00 | 0.00 | 0.00 | 0.00 | 0.24 | 0.43 | 0.01 | 0.10 | . | . |  |
| Feed_distributor | 0.00 | 0.00 | 0.00 | 0.00 | . | . | 0.05 | 0.22 | 0.26 | 0.44 |  |
| Middlemen | 0.01 | 0.10 | 0.00 | 0.00 | . | . | . | . | . | . |  |
| Market | . | . | . | . | 0.06 | 0.23 | . | . | . | . |  |
| Shop not agrovet | . | . | . | . | 0.01 | 0.07 | . | . | 0.20 | 0.40 |  |
| Never_purchased | 0.03 | 0.16 | 0.01 | 0.11 | 0.00 | 0.00 | . | . | 0.02 | 0.13 |  |
| N | 110 |  | 76 |  | 195 |  | 198 |  | 288 |  | 887 |

**Table 12. Types of information exchanged during purchasing of antimicrobials at agrovet shops.**

| Agrovet Interactions | (% of HHs reporting) | N |
| --- | --- | --- |
| I know what I need and I just tell them the medicine name and they don’t give me instructions | 25 | 217 |
| I know what I need and I just tell the them the medicine name and they give me instructions | 5 | 43 |
| I tell them symptoms of my animals and they tell me the antibiotic I need but no instructions | 29 | 251 |
| I tell them the symptoms of my animals and they tell me the antibiotic I need and instructions | 38 | 329 |
| I never purchase antibiotics | 3 | 26 |

**Table 13. Frequency of having a prescription before purchasing antibiotics.**

| **When you buy antibiotics, do you have a prescription?** | **Frequency** | **Percentage (%)** |
| --- | --- | --- |
| **Ghana** | | |
| never/rarely | 44 | 40.00 |
| sometimes | 28 | 25.45 |
| almost always | 38 | 34.55 |
| **Total:** | **110** | **100** |
| **Kenya** | | |
| never/rarely | 20 | 26.32 |
| sometimes | 6 | 7.89 |
| almost always | 50 | 65.79 |
| **Total:** | **76** | **100** |
| **Tanzania** | | |
| never/rarely | 191 | 97.95 |
| sometimes | 4 | 2.05 |
| **Total:** | **195** | **100** |
| **Zambia** | | |
| never/rarely | 158 | 79.80 |
| sometimes | 20 | 10.10 |
| almost always | 20 | 10.10 |
| **Zimbabwe** | | |
| never/rarely | 267 | 92.71 |
| sometimes | 17 | 5.90 |
| almost always | 4 | 1.39 |
| **Total:** | **288** | **100** |

**Table 14. Reported increase in treatment failure from antimicrobials**

| **Country** | **mean** | **sd** | **N** |
| --- | --- | --- | --- |
| Ghana | 0.61 | 0.49 | 109 |
| Kenya | 0.62 | 0.49 | 76 |
| Tanzania | 0.68 | 0.47 | 195 |
| Zambia | 0.22 | 0.42 | 198 |
| Zimbabwe | 0.72 | 0.45 | 288 |

**Table 15. Sources of animal health advice by country.** Advice was a categorical value with 0=none/rarely, 1=sometimes, 2=almost always. **Bolded text are variables included in models**

|  | **Ghana** | | **Kenya** | | **Tanzania** | | **Zambia** | | **Zimbabwe** | |
| --- | --- | --- | --- | --- | --- | --- | --- | --- | --- | --- |
| **Advice sources** | mean | sd | mean | sd | mean | sd | mean | sd | Mean | sd |
| **friends** | 0.92 | 0.76 | 0.70 | 0.85 | 1.73 | 0.56 | 0.69 | 0.80 | 1.03 | 0.74 |
| **agrovet** | 0.66 | 0.69 | 1.66 | 0.76 | 0.43 | 0.66 | 0.58 | 0.73 | 0.68 | 0.74 |
| feed | 0.30 | 0.55 | 0.45 | 0.72 | 0.10 | 0.33 | 0.15 | 0.43 | 0.55 | 0.69 |
| Extension worker | 0.72 | 0.78 | 0.33 | 0.72 | 0.45 | 0.56 | 0.01 | 0.07 | 0.73 | 0.78 |
| **Government veterinarian** | 0.95 | 0.89 | 0.33 | 0.72 | 0.75 | 0.58 | 0.28 | 0.55 | 0.65 | 0.75 |
| **Private veterinarian** | 0.31 | 0.63 | 0.72 | 0.90 | 0.75 | 0.58 | 0.22 | 0.61 | 0.06 | 0.28 |
| **Laboratory personnel** | 0.06 | 0.28 | 0.00 | 0.00 | 0.07 | 0.27 | 0.05 | 0.25 | 0.10 | 0.35 |
| N | 110 |  | 76 |  | 195 |  | 198 |  | 288 |  |

**Table 16. People administering antibiotics by country.** Treatment is a categorical variable with 0=never/rarely, 1=sometimes, 2=almost always.

|  | **Ghana** | | **Kenya** | | **Tanzania** | | **Zambia** | | **Zimbabwe** | |
| --- | --- | --- | --- | --- | --- | --- | --- | --- | --- | --- |
| **Drug administrator** | mean | sd | mean | sd | mean | sd | mean | sd | Mean | sd |
| Farm owner | 1.13 | 0.91 | 1.68 | 0.73 | 0.91 | 0.28 | 1.41 | 0.84 | 1.61 | 0.69 |
| Friends_ | 0.26 | 0.50 | 0.54 | 0.76 | 0.16 | 0.37 | 0.36 | 0.64 | 0.75 | 0.65 |
| Farm manager | 1.27 | 0.87 | 0.21 | 0.62 | . | . | 0.29 | 0.67 | . | . |
| Agrovet | 0.05 | 0.27 | 0.09 | 0.33 | 0.03 | 0.16 | 0.58 | 0.74 | 0.12 | 0.37 |
| Feed company | 0.01 | 0.10 | 0.03 | 0.23 | . | . | 0.01 | 0.07 | 0.08 | 0.33 |
| Extension | 0.05 | 0.21 | 0.03 | 0.23 | 0.02 | 0.12 | 0.00 | 0.00 | 0.40 | 0.67 |
| Government veterinarian | 0.47 | 0.71 | 0.08 | 0.32 | 0.06 | 0.24 | 0.16 | 0.44 | 0.30 | 0.61 |
| Private veterinarian | 0.15 | 0.47 | 0.08 | 0.32 | 0.06 | 0.24 | 0.02 | 0.16 | 0.03 | 0.21 |
| Laboratory personnel | 0.00 | 0.00 | 0.49 | 0.74 | 0.01 | 0.07 | 0.00 | 0.00 | 0.06 | 0.28 |
| N | 110 |  | 76 |  | 195 |  | 198 |  | 288 |  |

**Table 17. Variables in KAP measures by country. Knowledge items were binary coded (1=Yes, 0=No) with 1 indicating correct.** Attitudes and practices were on a three-point Likert scale (0=none/rarely, 1=sometimes, 2=almost always).

| **Knowledge** | **Ghana** | | **Kenya** | | **Tanzania** | | **Zambia** | | **Zimbabwe** | |
| --- | --- | --- | --- | --- | --- | --- | --- | --- | --- | --- |
|  | mean | sd | mean | sd | mean | sd | mean | sd | mean | sd |
| Know AMR | 0.32 | 0.47 | 0.61 | 0.49 | 0.05 | 0.21 | 0.40 | 0.49 | 0.28 | 0.45 |
| Know Residues | 0.50 | 0.50 | 0.58 | 0.50 | 0.28 | 0.45 | 0.36 | 0.48 | 0.56 | 0.50 |
| Know Withdrawal | 0.82 | 0.39 | 0.47 | 0.50 | 0.50 | 0.50 | 0.63 | 0.48 | 0.84 | 0.37 |
| Knowl Vaccines | 0.98 | 0.13 | 0.99 | 0.11 | 0.69 | 0.46 | 0.97 | 0.17 | 0.64 | 0.48 |
| Told of AMR | 0.52 | 0.50 | 0.53 | 0.50 | 0.60 | 0.49 | 0.49 | 0.50 | 0.64 | 0.48 |
| **Attitudes** | mean | sd | mean | sd | mean | sd | mean | sd | mean | sd |
| Too many AMs limits effectivness | 0.71 | 0.46 | 0.87 | 0.34 | 0.10 | 0.30 | 0.78 | 0.41 | 0.65 | 0.48 |
| AMs can prevent disease | 0.38 | 0.49 | 0.38 | 0.49 | 0.07 | 0.26 | 0.48 | 0.50 | 0.22 | 0.41 |
| AMs can help growth | 0.47 | 0.50 | 0.57 | 0.50 | 0.83 | 0.38 | 0.63 | 0.48 | 0.61 | 0.49 |
| Important to consult vet | 0.88 | 0.32 | 0.93 | 0.25 | 0.82 | 0.38 | 0.65 | 0.48 | 0.97 | 0.18 |
| Vaccines reduces AMs | 0.54 | 0.50 | 0.76 | 0.43 | 0.68 | 0.47 | 0.65 | 0.48 | 0.67 | 0.47 |
| Observing withdrawal important | 0.74 | 0.44 | 0.71 | 0.46 | 0.46 | 0.50 | 0.93 | 0.26 | 0.88 | 0.32 |
| **Practices** | mean | sd | mean | sd | mean | sd | mean | sd | mean | sd |
| Consume products within withdrawal period | 0.25 | 0.43 | 0.61 | 0.49 | 0.15 | 0.36 | 0.96 | 0.20 | 0.94 | 0.24 |
| Sell products within withdrawal period | 0.07 | 0.26 | 0.13 | 0.34 | 0.98 | 0.12 | 0.98 | 0.12 | 1.00 | 0.00 |
| Give products to animals within withdrawal period | 1.00 | 0.00 | 0.84 | 0.37 | 0.92 | 0.28 | 0.98 | 0.12 | 0.97 | 0.18 |
| Use a larger dose of AMs | 0.83 | 0.38 | 0.96 | 0.20 | 0.64 | 0.48 | 0.95 | 0.21 | 0.94 | 0.24 |
| Use a smaller dose of AMs | 0.94 | 0.25 | 0.96 | 0.20 | 0.80 | 0.40 | 0.93 | 0.26 | 0.91 | 0.29 |
| Stop treat if animal improves | 0.70 | 0.46 | 0.88 | 0.33 | 0.50 | 0.50 | 0.89 | 0.31 | 0.80 | 0.40 |
| Use expired medicines | 0.97 | 0.16 | 0.99 | 0.11 | 0.78 | 0.41 | 0.98 | 0.14 | 0.97 | 0.17 |
| Have a prescription for AM | 0.35 | 0.48 | 0.66 | 0.48 | 0.00 | 0.00 | 0.10 | 0.30 | 0.01 | 0.12 |
| Observe withdrawal from antibiotics | 0.78 | 0.41 | 0.87 | 0.34 | 0.64 | 0.48 | 0.84 | 0.37 | 0.90 | 0.31 |
| N | 110 |  | 76 |  | 195 |  | 198 |  | 288 |  |

**Figure 6. One-Way ANOVA results for percent of KAP knowledge correct and Tukey-Hamer pairwise tests.** For Tukey-Hamer comparisons: 1 = Ghana, 2 = Kenya, 3= Tanzania, 4= Zambia, 5 = Zimbabwe.


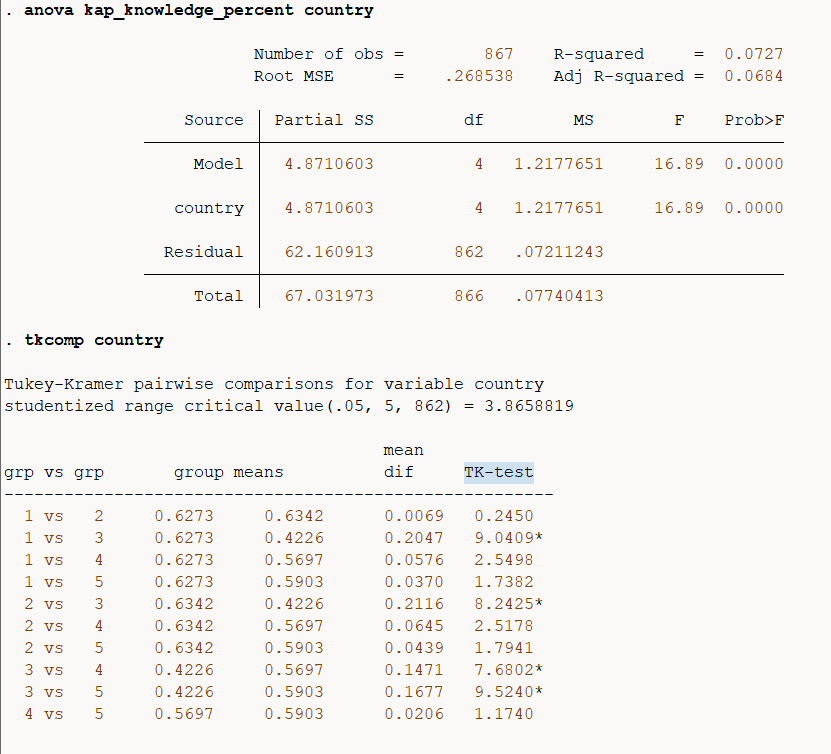


**Figure 7. One-Way ANOVA results for percent of desirable KAP attitudes and Tukey-Hamer pairwise tests.** For Tukey-Hamer comparisons: 1 = Ghana, 2 = Kenya, 3= Tanzania, 4= Zambia, 5 = Zimbabwe.


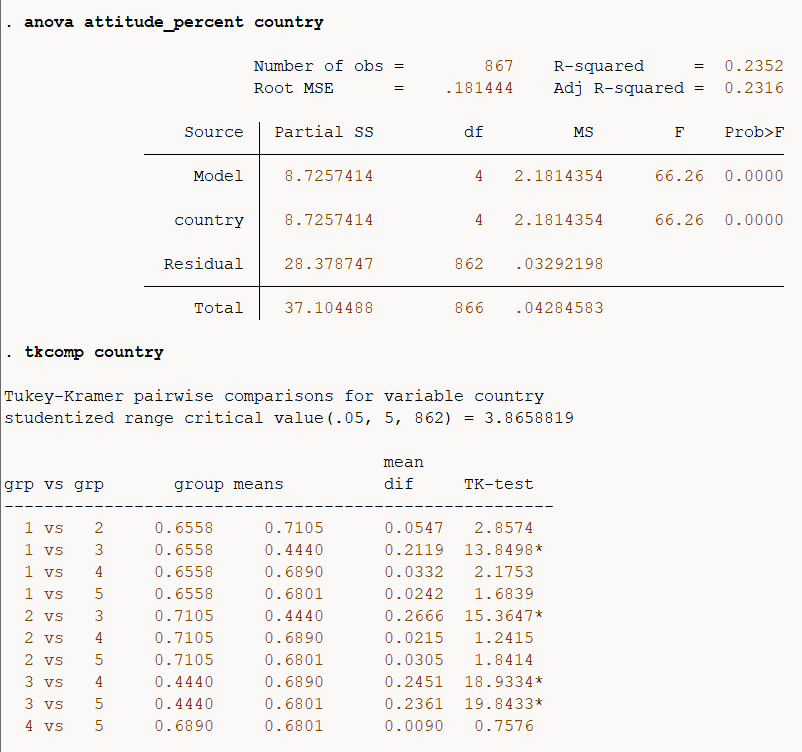


**Figure 8. One-Way ANOVA results for percent of prudent practices and Tukey-Hamer pairwise tests.** For Tukey-Hamer comparisons: 1 = Ghana, 2 = Kenya, 3= Tanzania, 4= Zambia, 5 = Zimbabwe.


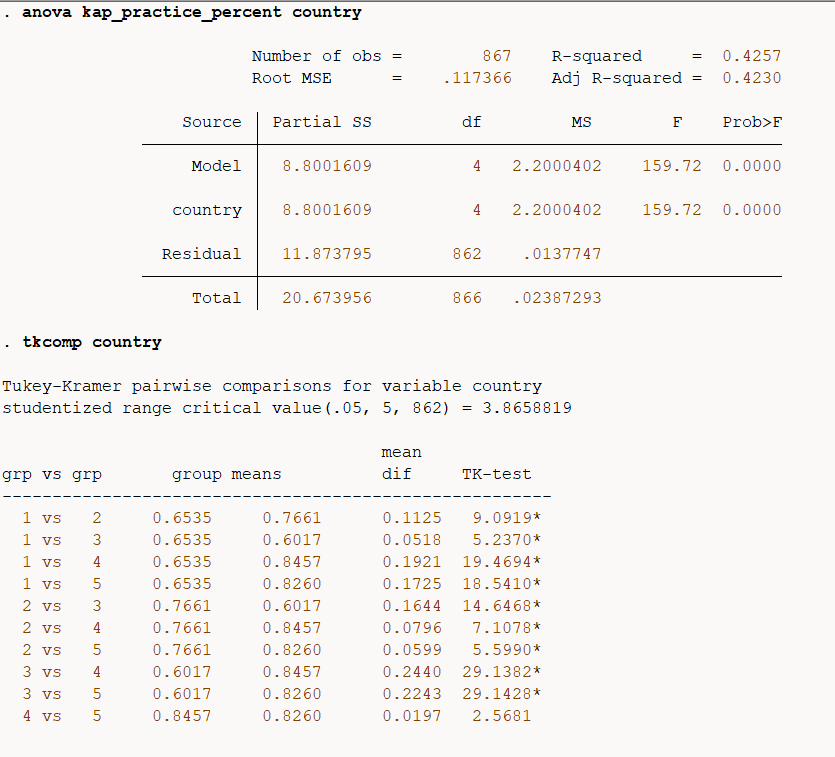


**References**

1. Mbugua MW. Analysis Of Demand For Antibiotics In Poultry Production In Kiambu County, Kenya. Thesis. Nairobi Kenya: University of Nairobi, Department of Agricultural Economics; 2014. http://erepository.uonbi.ac.ke/handle/11295/71821. Accessed 11 Sep 2018.

2. Adelaide O, Bii C, Okemo P. Antibiotic resistance and virulence factors in Escherichia coli from broiler chicken slaughtered at Tigoni processing plant in Limuru, Kenya. East Afr Med J. 2008;85:597–606.

3. Bernsten JL. The Maasai and their Neighbors: variables of interaction. Afr Econ Hist. 1976;2:1–11.

4. Fratkin E. East African Pastoralism in Transition: Maasai, Boran, and Rendille Cases. Afr Stud Rev. 2001;44:1–25.

5. Galaty JG, editor. Maasai expansion and the new East African pastoralism. Athens, OH: Ohio University Press; 1993.

6. Hodgson DL. Once intrepid warriors: Gender, ethnicity, and the cultural politics of Maasai development. Bloomington: Indiana University Press; 2001.

7. Spear T, Waller R. Being Maasai: ethnicity and identity in East Africa. Athens, OH: Ohio University Press; 1993.

8. Baird TD, Gray CL. Livelihood Diversification and Shifting Social Networks of Exchange: A Social Network Transition? World Dev. 2014;60:14–30.

9. McCabe JT. Cattle bring us to our enemies. Ann Arbor, MI: University of Michigan Press Ann Arbor; 2004.

10. McCabe JT, Leslie PW, DeLuca L. Adopting Cultivation to Remain Pastoralists: The Diversification of Maasai Livelihoods in Northern Tanzania. Hum Ecol. 2010;38:321–34.

11. Msoffe FU, Kifugo SC, Said MY, Neselle MO, Van Gardingen P, Reid RS, et al. Drivers and impacts of land-use change in the Maasai Steppe of northern Tanzania: an ecological, social and political analysis. J Land Use Sci. 2011;6:261–81.

12. Sangeda AZ, Maleko DD. Rangeland condition and livestock carrying capacity under the traditional rotational grazing system in northern Tanzania. Livest Res Rural Dev. 2018;30:5.

13. Creswell JW, Clark VLP. Designing and conducting mixed methods research. Third. Thousand Oaks, CA: Sage publications; 2017.

14. Bernard HR. Research methods in anthropology: Qualitative and quantitative approaches. Fourth. Walnut Creek, Calif.: Altamira Press; 2011.

15. Vaismoradi M, Turunen H, Bondas T. Content analysis and thematic analysis: Implications for conducting a qualitative descriptive study. Nurs Health Sci. 2013;15:398–405.

16. Caudell MA, Quinlan MB, Subbiah M, Call DR, Roulette CJ, Roulette JW, et al. Antimicrobial Use and Veterinary Care among Agro-Pastoralists in Northern Tanzania. PloS One. 2017;12:e0170328.
