# Supplementary material for: Towards a bottom-up understanding of antimicrobial use and resistance on the farm: A knowledge, attitudes, and practices survey across livestock systems in five African countries
Source: PLoS One. 2020 Jan 24;15(1):e0220274. doi: 10.1371/journal.pone.0220274 (PMC6980545; doi:10.1371/journal.pone.0220274)
Supplement: S1 File — (PDF) [file pone.0220274.s002.pdf]

Zambia_KAP_Finalized

Study Introduction

Hello, my name is __ and I am working with the Ministry of Fisheries and Livestock with support from Food and Agricultural Organization on a project looking at poultry farmers with focus on broiler production in Zambia.

We are interested in knowing how you manage your farm and we want to know this because we want to develop a program to help you improve the health of your animals

This survey will take around 1 hr of your time

Your participation is voluntary and you can stop at any time you choose to and there will be no negative consequences for you

Anything you say during the discussion today will remain completely confidential: your name will not be used in any materials produced from this study nor any other information that could be used to identify you

You can request access to, moderations to, and/or deletion of your personal data.

Informed Consent

The study has been explained to me in a language that I comprehend. All the questions I had about the study have been answered.

I have been informed that it is my RIGHT TO REFUSE to participate today and that if I choose to refuse I do not have to give a reason, and there will be NO NEGATIVE CONSEQUENCES for me.

I have been informed that anything I say during the discussion today will remain COMPLETELY CONFIDENTIAL: my name will not be used in any materials produced from this study nor any other information that could be used to identify me

I have been informed that I can request ACESSS TO, MODERATIONS TO, and/or DELETION of my personal data.

I release FAO and its employees dealing with the use of my personal data as described above.

I agree to take part of this study

- ◯ Yes
- ◯ No

Please have them sign

1 Please Check Name

- [redacted]
- [redacted]
- [redacted]
- [redacted]
- [redacted]
- [redacted]
- [redacted]
- [redacted]

2 Please Select District

- Chibombo
- Chisamba
- Chongwe
- Chilanga
- Rufunsa

3 Please select Camp

- ◯ Chukumbi
- ◯ Chibombo East Camp
- ◯ Chipembi
- ◯ Namalombwe
- ◯ Chongwe Central
- ◯ Chalimbawa
- ◯ Mwembeshi
- ◯ Ngawena

4 Respondent position in the household

- ◯ Farm Owner
- ◯ Farm Manager
- ◯ Farm Employee
- ◯ Household member

5 What type of relative?

- ◯ spouse
- ◯ son
- ◯ daughter
- ◯ brother
- ◯ sister
- ◯ mother
- ◯ cousin
- ◯ In law
- ◯ friend

6 Respondent Birth Year

____

7 Respondent Gender

- ◯ Male
- ◯ Female

8 A) Is the farm owned by an institution?

- ◯ Yes
- ◯ No

9 Farm owner Gender

- Male
- Female

10 Farm owner Birth Year

____

12 What level of formal education does the RESPONDENT have?

- None
- Uncompleted Primary Level
- Completed Primary Level
- Uncompleted Junior secondary level
- Completed Junior seconary level
- Uncompleted senior secondary level
- Completed senior secondary
- Certificate after Primary
- Diploma/Certificate after Secondary
- University Graduate
- Post-Graduate of University
- Don't Know

13 What level of formal education does the Farm Owner have?

- None
- Uncompleted Primary Level
- Completed Primary Level
- Uncompleted Junior secondary level
- Completed Junior seconary level
- Uncompleted senior secondary level
- Completed senior secondary
- Certificate after Primary
- Diploma/Certificate after Secondary
- University Graduate
- Post-Graduate of University
- Don't Know

14 Can you read?

- ◯ Yes
- ◯ No

15 How many people live on the farm?

____

16 How many employees does the farm have? (NOT HOUSEHOLD MEMBERS)

____

17 What is MAIN occupation of the farm owner?

- ◯ farmer
- ◯ small scale trade(kantemba)
- ◯ shop owner
- ◯ government worker
- ◯ formal worker
- ◯ informal worker
- ◯ Student
- ◯ No Occupation: Owner is Retired
- ◯ Other

Specify other.

____

18 Is farm under contract with an Outgrower?

- ◯ Yes
- ◯ No

19 Name of Outgrower(s)

____

20 Now, I will give you a list of animals. I will ask you to tell me how many are at the farm and whether they are in a pen or free-ranging

21 How many dairy cattle does the household have?

____

22 Are your dairy cattle?

- Free Range All DAY and ALL NIGHT
- Free Range All DAY and CONFINED AT NIGHT
- Confined All Day & All Night

23 How many beef cattle do you have?

____

24 Are your beef cattle?

- Free Range All DAY and ALL NIGHT
- Free Range All DAY and CONFINED AT NIGHT
- Confined All Day & All Night

25 How many sheep do you have?

____

26 Are your sheep?

- Free Range All DAY and ALL NIGHT
- Free Range All DAY and CONFINED AT NIGHT
- Confined All Day & All Night

27 How many goats do you have?

____

28 Are your goats?

- Free Range All DAY and ALL NIGHT
- Free Range All DAY and CONFINED AT NIGHT
- Confined All Day & All Night

29 How many pigs do you have?

____

30 Are your pigs?

- Free Range All DAY and ALL NIGHT
- Free Range All DAY and CONFINED AT NIGHT
- Confined All Day & All Night

31 How many dogs do you have?

____

32 How many donkeys do you have?

____

33 How many horses do you have?

____

34 How many rabbits do you have?

____

35 How many geese do you have?

____

36 Are your geese?

- ◯ Free Range All DAY and ALL NIGHT
- ◯ Free Range All DAY and CONFINED AT NIGHT
- ◯ Confined All Day & All Night

37 How many ducks do you have?

____

38 Are your ducks?

- ◯ Free Range All DAY and ALL NIGHT
- ◯ Free Range All DAY and CONFINED AT NIGHT
- ◯ Confined All Day & All Night

39 How many turkeys do you have?

____

40 Are your turkeys?

- ◯ Free Range All DAY and ALL NIGHT
- ◯ Free Range All DAY and CONFINED AT NIGHT
- ◯ Confined All Day & All Night

41 How many local chickens do you have?

____

42 Are your local chickens?

- Free Range All DAY and ALL NIGHT
- Free Range All DAY and CONFINED AT NIGHT
- Confined All Day & All Night

43 How many guinea fowls do you have?

____

44 Are your guinea fowls?

- Free Range All DAY and ALL NIGHT
- Free Range All DAY and CONFINED AT NIGHT
- Confined All Day & All Night

45 How many quails do you have?

____

46 Are your quails?

- Free Range All DAY and ALL NIGHT
- Free Range All DAY and CONFINED AT NIGHT
- Confined All Day & All Night

47 How many layers do you have?

____

48 Are your layers kept?

- Free Range All DAY and ALL NIGHT
- Free Range All DAY and CONFINED AT NIGHT
- Confined All Day & All Night

50 Now, I'd like to ask some questions specific to broilers

51 What is the MAIN reason you keep broilers?

- ◯ commercial(mostly for selling)
- ◯ Subsistence (own consumption)
- ◯ Hobby
- ◯ Prestige

52 How many birds do you have that are between 0 weeks and 2 weeks?

____

53 How many birds do you have that are over 2 weeks and under 5 weeks?

____

54 How many birds do you have that are 5 weeks and over?

____

55 Do you USUALLY keep multiple ages of broilers at the same time?

- ◯ Yes
- ◯ No

56 How are multiple ages kept?

- ◯ I keep multiple ages in separate houses
- ◯ I keep multiple ages in the same house but seperated by A BARRIER
- ◯ I keep multiple ages in the same house but with NO BARRIER

57 Do you keep other types of poultry in broiler houses?

- ◯ Yes
- ◯ No

58 If yes, what type of poultry?

- ☐ Layers
- ☐ Local chicken
- ☐ Improved indigenous chicken (astrolob, sasu)

60 Keeping Time

61 About how many years or months have you been keeping broilers?
ROUND UP!

____

62 Record whether years or months

- ◯ months
- ◯ years

64 Now, I'll ask you a few questions on production costs. ASK THEM FOR COSTS FOR LAST PRODUCTION CYCLE OR CURRENT PRODUCTION cycle if they have not completed a full cycle

65 Can you estimate the money the farm spent on medicine/vaccines/and disenfectants/litter/feed the last production cycle (or current production cycle)?

- ◯ Yes
- ◯ No

66 Could you provide an estimate of the following:

67 Lump sum costs of products to control disease during last production cycle (antibiotics/stresspack/vaccines) current production cycle if they have not completed a full cycle

In KWACHA. Write DK if DON"T KNOW

____

68 Lump sum costs of feed during last production cycle or current production cycle if they have not completed a full cycle

In KWACHA. Write DK if DON"T KNOW

____

69 Lump sum costs of litter during last production cycle or current production cycle if they have not completed a full cycle

In KWACHA. Write DK if DON"T KNOW

____

70 Lump sum costs of disinfectants during last production cycle or current production cycle if they have not completed a full cycle

In KWACHA. Write DK if DON"T KNOW

____

72 Who is the main breeder of the broiler you stock?

- ◯ Bokomo
- ◯ Hybrid
- ◯ Panda
- ◯ Ross
- ◯ Tiger feeds
- ◯ Yielding Tree
- ◯ Zam Chick
- ◯ Other

Specify other.

---

73 Where do you source your starter flock (day-old chicks) Select All that Apply

- ☐ Breeder
- ☐ Agrovet
- ☐ Other Farmers
- ☐ Trader/agent
- ☐ Other

Specify other.

---

74 Does your starter flock come vaccinated against some diseases?

- ◯ yes
- ◯ no
- ◯ Don't Know

75 When you go to the place where you get your starter flock, what else do you get? Select All that Apply

- [ ] Only chicks
- [ ] Feed
- [ ] Glucose
- [ ] information(vaccine schedule, general animal health
- [ ] Liquid Paraffin
- [ ] Medicine
- [ ] Multivitamins
- [ ] Vaccines
- [ ] Other

Specify other.

____

76 Now, I would like to ask you a few questions about the health of your poultry

77 Number of broilers that died during last production cycle or current production cycle if they have not completed a full cycle

An ESTIMATE is fine

____

78 What are the most common SYMPTOMS your broilers show? Please Select All that Apply
Symptoms listed in alphabetical order

- [ ] Ascites (accumulation of fluid)
- [ ] Blindness
- [ ] Bloody Droppings/Diarrhea
- [ ] Circling
- [ ] CNS disorders
- [ ] convulsion
- [ ] Coughing
- [ ] Diarrhea
- [ ] Discharge from Eyes
- [ ] Discoloration of the vent (green)
- [ ] Dullness
- [ ] Green diarrhea
- [ ] Lameness
- [ ] Lesions on skin
- [ ] mated feathers at the vent
- [ ] Nasal discharge
- [ ] Paralysis
- [ ] Pecking each other
- [ ] Sleepiness
- [ ] Sneezing
- [ ] Snoring
- [ ] stargazing
- [ ] Stunting
- [ ] Sudden Death
- [ ] Swollen Eyes
- [ ] swollen head
- [ ] Swollen Vent
- [ ] twisted neck / crazy neck
- [ ] White Diarrhea
- [ ] wizzing
- [ ] Other

Specify other.

---

79 What are the most common diseases your broilers get? Please Select All that Apply

- ☐ Coccidiosis
- ☐ Coryza
- ☐ CRD (Chronic Respiratory Disease)
- ☐ Fowl cholera
- ☐ Fowl Pox
- ☐ Fowl typhoid
- ☐ Gumboro Disease
- ☐ Infectious Bronchitis
- ☐ Marek's Disease
- ☐ Newcastle Disease (Chigodora/chipumpu)
- ☐ Pulmonary Hypertension Syndrome
- ☐ Sudden Death Syndrome
- ☐ Worms
- ☐ Ectoparasites
- ☐ I don't know any diseases
- ☐ My birds never get disease
- ☐ Other

Specify other.

---

80 What are the most common SYMPTOMS your local chickens show? Please Select All that Apply

Symptoms listed in alphabetical order

- [ ] Ascites (accumulation of fluid)
- [ ] Blindness
- [ ] Bloody Droppings/Diarrhea
- [ ] Circling
- [ ] CNS disorders
- [ ] convulsion
- [ ] Coughing
- [ ] Diarrhea
- [ ] Discharge from Eyes
- [ ] Discoloration of the vent (green)
- [ ] Dullness
- [ ] Green diarrhea
- [ ] Lameness
- [ ] Lesions on skin
- [ ] mated feathers at the vent
- [ ] Nasal discharge
- [ ] Paralysis
- [ ] Pecking each other
- [ ] Sleepiness
- [ ] Sneezing
- [ ] Snoring
- [ ] stargazing
- [ ] Stunting
- [ ] Sudden Death
- [ ] Swollen Eyes
- [ ] swollen head
- [ ] Swollen Vent
- [ ] twisted neck / crazy neck
- [ ] White Diarrhea
- [ ] wizzing
- [ ] Other

Specify other.

____

81 What are the most common diseases your local chickens get? Please Select All that Apply

- [ ] Coccidiosis
- [ ] Coryza
- [ ] CRD (Chronic Respiratory Disease)
- [ ] Fowl cholera
- [ ] Fowl Pox
- [ ] Fowl typhoid
- [ ] Gumboro Disease
- [ ] Infectious Bronchitis
- [ ] Marek's Disease
- [ ] Newcastle Disease (Chigodora/chipumpu)
- [ ] Pulmonary Hypertension Syndrome
- [ ] Sudden Death Syndrome
- [ ] Worms
- [ ] Ectoparasites
- [ ] I don't know any diseases
- [ ] My birds never get disease
- [ ] Other

Specify other.

____

82 Where do you MOSTLY get information/advice on disease treatment for your sick broilers?

- ◯ Agrovet
- ◯ Breeders of broilers
- ◯ Family
- ◯ Farm manager
- ◯ Farm owner
- ◯ Feed Distributor
- ◯ Government vet services
- ◯ Other farmers
- ◯ Private vet services
- ◯ Veterinary laboratory
- ◯ I never get advice because I know what to do
- ◯ Other

Specify other.

---

84 Bird Health

85 How often do these people give you advice/instructions on bird health (GIVE THEM OPTIONS OF NEVER, RARELY, SOMETIMES, ALMOST ALWAYS)	never	rarely	sometimes	almost always
86 Family/friends	◯	◯	◯	◯
87 NGOs	◯	◯	◯	◯
88 agrovet	◯	◯	◯	◯
89 feed distributor	◯	◯	◯	◯
90 a government veterinarian	◯	◯	◯	◯
91 a government para veterinarian	◯	◯	◯	◯
92 a private para veterinarian	◯	◯	◯	◯
93 a private veterinarian	◯	◯	◯	◯
94 a vet laboratory	◯	◯	◯	◯
95 community animal health worker	◯	◯	◯	◯
96 Breeder of Broilers	◯	◯	◯	◯

98 How often do you take samples/sick birds to a veterinarian/para veterinarian/laboratory for diagnosis?

- ○ never
- ○ rarely
- ○ sometimes
- ○ always whenever disease strikes

99 What challenges do you face in trying to get veterinary health services?LIST ALL and SELECT ALL that Apply

- ☐ High Cost
- ☐ Distance
- ☐ Too few services
- ☐ Lack of follow-up
- ☐ Existence of Quacks
- ☐ SLOW response time/turn around
- ☐ NO RESPONSE
- ☐ No challenges
- ☐ Other

Specify other.

____

100 What challenges do you face in trying to get diagnosis at a veterinary laboratory? LIST ALL and SELECT ALL that Apply

- ☐ High Cost
- ☐ Distance to Lab
- ☐ Not aware of lab
- ☐ Don't know what to take to lab
- ☐ Samples taken and no response
- ☐ Slow Services
- ☐ No challenges
- ☐ DOES NOT APPLY: I DO NOT TAKE SAMPLES
- ☐ Other

Specify other.

____

101 How many kilometers away is the agroshop you use?

____

102 Are there some medicines that used to work in the past that are not as effective at treating diseases in the present?

including acaracides

- ◯ yes
- ◯ no
- ◯ I don't know

103 What medicine(s)?

____

104 Why do you believe the medicines are less effective?

____

105 What do you do when you observe that the medicines you used are no longer effective? Select all that Apply

- ☐ Try a different drug
- ☐ Increase drug dosage
- ☐ Combine with other drugs
- ☐ Consult with animal health professional
- ☐ Shift to herbal/traditional medicine

106 Can you explain what antibiotics do? (DO NOT LIST BUT Select All That Apply)

provide an example of an antibioticsuch as tetracycline

- ☐ medicine
- ☐ medicine that kills disease
- ☐ medicine that kills germs
- ☐ medicines that kill bacteria
- ☐ medicine that PREVENTS diseases
- ☐ medicines that PROMOTE GROWTH
- ☐ medicine increases EGG LAYING
- ☐ medicine that KILLS VIRUSES
- ☐ Doesn't know
- ☐ Other

Specify other.

---

107 Where do you USUALLY get your antibiotics from? Select One
Provide an example of an antibiotic such as TETROXY if needed

- ◯ Breeder
- ◯ Feed distributor
- ◯ Friend
- ◯ Agrovet shop
- ◯ Community health care worker
- ◯ Pharmacy/Drug shops
- ◯ Government vet/paravet
- ◯ Illegal briefcase dealers
- ◯ I've never purchased antibiotics
- ◯ NGOs
- ◯ private vet/paravet
- ◯ Other

Specify other.

---

108 When you buy antibiotics, do you usually have a prescription, meaning an official piece of paper given to you by a qualified animal health professional, or does veterinarian bring antibiotics?

- ◯ never
- ◯ rarely
- ◯ sometimes
- ◯ almost always

109 Why did you usually not have a presciption?

---

110 When you buy antibiotics does the seller USUALLY ask you to have a prescription?

- ◯ never
- ◯ rarely
- ◯ sometimes
- ◯ almost always

111 Does the seller USUALLY give you instructions on using AMS?

- ◯ No, I just tell him what I need and they give me the drug
- ◯ Yes, everytime I get drug they tell me instructions
- ◯ Sometimes they give me instructions

112 Think about your broilers, about how many days are they given something with antibiotics in it during one production cycle?

Remember, this is an estimate. If they say "half of days" put 21

____

113 Think about ALL YOUR ANIMALS how often were they given antibiotics in the last ONE month?

- ◯ Never
- ◯ One or Two
- ◯ 3 to 5 times
- ◯ 6 to 10 times
- ◯ Over 10 times

114 Think about YOUR FAMILY, how often have they taken antibiotics in the last ONE month?

- ◯ Never
- ◯ One or Two
- ◯ 3 to 5 times
- ◯ 6 to 10 times
- ◯ Over 10 times

115 Please list all the reasons you use antibiotics on your animals? Please Select All that Apply

cirprofloxin, penicillin, amoxycillin, tertracycline

- ☐ To help sick animals get better
- ☐ To PREVENT A GROUP OF ANIMALS from getting sick from other animals
- ☐ To PREVENT A SINGLE ANIMAL from getting sick
- ☐ To help animals GROW FASTER
- ☐ To help animals GROW BIGGER
- ☐ To help birds LAY MORE EGGS
- ☐ I don't use antibiotics on animals
- ☐ Other

Specify other.

____

116 Now, FOR YOUR BIRDS, please think about when give antibiotics

**117 How many of these people give medicine at your farm? (GIVE THEM OPTIONS OF NEVER, RARELY, SOMETIMES, ALMOST ALWAYS)**	never	rarely	sometimes	almost always
**118 Farm Owner**	◯	◯	◯	◯
**119 Family or Friends of Farm Owners**	◯	◯	◯	◯
**120 Farm Manager**	◯	◯	◯	◯
**121 Farm Worker**	◯	◯	◯	◯
**122 Breeders/Feed Company Worker**	◯	◯	◯	◯
**123 General Agriculutral Extension Worker**	◯	◯	◯	◯
**124 Community Animal Health Worker**	◯	◯	◯	◯
**125 Govt. Vet**	◯	◯	◯	◯
**126 Priv. Vet**	◯	◯	◯	◯
**127 Vet lab worker**	◯	◯	◯	◯

129 Do you give any medication to your day-old chicks?

- ◯ never
- ◯ rarely
- ◯ sometimes
- ◯ almost always

130 What types of medication do you give?

- ☐ Stresspack
- ☐ Tetroxy
- ☐ Boosters
- ☐ Tetravit

131 When a few of your birds get sick do you usually give medicine to all of them (for example, in a drinker)?

- ◯ never
- ◯ rarely
- ◯ sometimes
- ◯ almost always

132 Do you use an isolation pen/house when a few of your birds are sick?

- yes, I keep sick birds in a different room
- yes, I keep sick birds in same room but with a barrier
- no

133 Do you use human medicines to treat your birds?

- never
- sometimes
- almost always
- don't know

134 What medicines are they?

---

135 How do you USUALLY measure the quantity of medicine to give to birds?

- Spoons
- Bottle cap
- Calibrated cups
- Syringes
- Scale
- Use the entire satchet with precribed amount of water

136 Do you ever give birds a LARGER DOSE than the recommended dose?

- never
- rarely
- sometimes
- almost always

137 Briefly record WHY they DO give a larger dose

---

138 Do you ever give birds a SMALLER DOSE than the recommended dose?

- never
- rarely
- sometimes
- almost always

139 Briefly record WHY they DO give a smaller dose

---

140 Do you ever STOP using medicine before the full dose?

- ◯ never
- ◯ rarely
- ◯ sometimes
- ◯ almost always

141 Briefly record WHY they STOP treatment

---

142 Do you regulary check the expiration data of drugs?

- ◯ never
- ◯ rarely
- ◯ sometimes
- ◯ almost always

143 If you find that they are expired can you use them?

- ◯ Yes
- ◯ No

144 Why can you use expired drugs?

---

145 If the antibiotics you have become expired, what do you USUALLY do with them?

- ◯ I do not worry about the expiry date
- ◯ I've never had drugs expire
- ◯ Return them back to where you bought them
- ◯ Throw away in compost pit
- ◯ Throw away in latrine
- ◯ Throw away in refuse pit
- ◯ Will not use them and bury
- ◯ Will use them until they are finished
- ◯ Other

Specify other.

---

146 When a bird has died, what do you USUALLY do with it?

- ◯ Burn it
- ◯ Bury it
- ◯ Consume at home
- ◯ Eat it
- ◯ Give it to dogs
- ◯ Sell it
- ◯ Share with neighbors and friends
- ◯ Throw it in a pit latrine
- ◯ Throw it in a refuse pit
- ◯ Other

Specify other.

---

147 Have you ever heard about "antibiotic resistance" or "antimicrobial resistance"?

- ◯ Yes
- ◯ No

148 How much do you believe antibiotic or antimicrobial resistance will impact you, your family/friends, and your animals? LIST THE OPTIONS

- ◯ no impact
- ◯ a little impact
- ◯ a large impact
- ◯ I don't know the level of impact

149 HOW do you think antibiotic/antimicrobial resistance will impact your livelihood?

- ☐ Loss of animals/money from farming
- ☐ Loss of human life (you, your family, your friends, your community)
- ☐ I don't know
- ☐ Other

Specify other.

---

150 Who told you about antibiotic/antimicrobial resistance?

- [ ] Agrodealer Employee
- [ ] Breeder
- [ ] Clinic
- [ ] Extension worker
- [ ] Family Member
- [ ] Farm Manager
- [ ] Feed Company Person
- [ ] Friend or Neighbor
- [ ] Govt. Vet
- [ ] I don't remember where I heard it
- [ ] Learned from school
- [ ] Other Internet Sites
- [ ] Print Media
- [ ] Priv. Vet
- [ ] Radio
- [ ] Social Media: Facebook
- [ ] Social Media: WhatsApp
- [ ] TV
- [ ] Vet lab worker
- [ ] Other

Specify other.

____

151 NOW, I WILL READ YOU A SET OF STATEMENTS AND ASK YOU HOW MUCH YOU AGREE WITH THEM. I'll ask whether you agree, neither agree nor disagree, or disagree

153 You can stop giving an animal a full course of antibiotic if their symptoms are improving.

- ○ agree
- ○ disagree
- ○ I don't know

154 If medicines are given TOO OFTEN then they might stop working.

- agree
- disagree
- I don't know

155 Giving animals that are not sick antibiotcs will PREVENT THEM FROM BECOMING SICK in the future.

- agree
- disagree
- I don't know

156 Giving animals antibiotics can help them GROW BIGGER, FASTER, FATTER, boost egg production/size.

- agree
- disagree
- I don't know

157 It is important to get CONSULTATION FROM A VETERINARIAN before giving antibiotics to the animals.

- agree
- disagree
- I don't know

158 After using antibiotics on an animal, you should WAIT sometime before you sell or consume the PRODUCTS from it, such as bird meat/eggs/milk.

- agree
- disagree
- I don't know

160 Can animals and animal products from sick animals transmit disease to humans?

- yes
- no
- Don't Know

161 Do you know what antibiotic residues are?

- Yes
- No

162 What do you USUALLY do with the chickens under CURRENT TREATMENT with antibiotics/antimicrobials or during WITHDRAWAL PERIOD?

- use for home consumption
- Give to other animals
- Sell it
- Throw it away
- Wait for treatment to finish before selling or eating
- Wait for withdrawal period to finish before selling or eating

163 Has an animal health worker/poultry drug shop/veterinarian/NGO ever told you about withdrawal periods: that is, you have to wait to sell the animal or eat a meat/egg/milk after giving the animal medicine?

- Yes
- No

164 Now, I'd like to ask some questions about vaccines

165 Do you know what vaccines (Katemela are?)

If they say something like "PREVENT DISEASE LONG TERM" check yes

- Yes
- No

165A Do you vaccinate your birds?

- Yes
- No

165B Why do they not vaccinate?

166 What vaccines do you give to your broilers? Please Select All that Apply

- [ ] Coccidiosis
- [ ] Newcastle
- [ ] Gumboro
- [ ] Fowl Pox
- [ ] Fowl typhoid
- [ ] Marek's
- [ ] Ascites (accumulation of fluid)
- [ ] Infectious Bronchitis
- [ ] Coryza
- [ ] CRD (Chronic Respiratory Disease)
- [ ] Egg Perinoitis
- [ ] Other

Specify other.

---

167 What vaccines do you give to your OTHER DOMESTICATED BIRDS? Please Select All that Apply

- [ ] Coccidiosis
- [ ] Newcastle Diseases
- [ ] Gumboro Disease
- [ ] Fowl Pox
- [ ] Fowl typhoid
- [ ] Marek's Disease
- [ ] Ascites
- [ ] Infectious Bronchitis
- [ ] Coryza
- [ ] CRD (Chronic Respiratory Disease)
- [ ] Egg Perinoitis
- [ ] I don't know
- [ ] I ONLY HAVE BROILERS NOT ANY OTHER BIRDS
- [ ] Other

Specify other.

---

168 Have your broilers ever suffered from the disease they have been vaccinated for?

- ◯ never
- ◯ rarely
- ◯ sometimes
- ◯ almost always

169 Where do you purchase your vaccines from? Please Select All that Apply

- ☐ agrovet
- ☐ Breeders
- ☐ farmer associations
- ☐ feed distributor
- ☐ friends
- ☐ government program
- ☐ government vet
- ☐ Import from outside Zambia
- ☐ Pharmaceutical company
- ☐ private vet

170 Who USUALLY vaccinates the birds or broilers if vaccination?

- ◯ Agrovet
- ◯ Community health care worker
- ◯ extension worker (crop/livestock)
- ◯ family/friends
- ◯ Govt. Vet
- ◯ Myself
- ◯ Priv. Vet

171 How do you USUALLY transport your vaccines to the farm?

- ◯ Cooler box with ice pack
- ◯ Cooler box WITHOUT ice pack
- ◯ Flasks with ice pack
- ◯ In a plastic/box with ice cubes
- ◯ In a plastic/box WITHOUT ice
- ◯ Roof warmers with ice packs
- ◯ Vaccine corner cold box (WRONG WORDING)

172 How do you USUALLY store your vaccines at the farm?

- ◯ At room temperature on shelves
- ◯ Bucket of water
- ◯ Freezer
- ◯ Immediate Use
- ◯ Store in the refridgerator
- ◯ Under moist soil

173 What do you USUALLY do with extra vaccine?

- ◯ Discard it
- ◯ I always buy exact dose
- ◯ share it with another farmer
- ◯ store to use during the next vaccination
- ◯ use all of it

174 What challenges do you normally face with getting vaccinations? Please LIST ALL and SELECT ALL that Apply

- ☐ Unavailability
- ☐ High Cost of professional administration
- ☐ lack of information on what vaccines and when to use (don't always know where and how to get them)
- ☐ High cost of vaccines
- ☐ Far distance to purchase vaccines
- ☐ Don't have access to proper storage/handling
- ☐ Lack of appropriate doses
- ☐ No challenges
- ☐ Other

Specify other.

____

175 How much do you agree with the statement that "using vaccines can prevent the use of antibiotics?"

- ◯ agree
- ◯ ndipakati (I'm in the middle)
- ◯ disagree
- ◯ Respondent does not know the difference between vaccine and antibiotics

177 Now, I'd like to ask you a few questions about your broiler houses.

178 Record the number of broiler houses

____

179 dimension

180 Record the LENGTH of the house in meters

____

181 Record the WIDTH of the house in meters

____

183 How many birds are in the main broiler house?

____

184 How many weeks are the birds in the main broiler house?

- ◯ Under 1 week
- ◯ 1 week
- ◯ 2 weeks
- ◯ 3 weeks
- ◯ 4 weeks
- ◯ 5 weeks
- ◯ 6 weeks
- ◯ 7 weeks
- ◯ Over 8 weeks
- ◯ Not keeping broilers currently

185 Do you buy litter BEFORE bringing home chicks?

- ◯ never
- ◯ rarely
- ◯ sometimes
- ◯ almost always

186 Do you remove litter and disenfect house at least once during the cycle?

- ◯ Yes
- ◯ No

187 Do you treat your litter before putting chicks into the broiler house?

- ◯ never
- ◯ rarely
- ◯ sometimes
- ◯ almost always

188 What do you USUALLY use to treat your litter? Select All that Apply

- ◯ disinfectant
- ◯ Sun Drying
- ◯ Other

Specify other.

---

189 What do you do with the used litter? Please Select All that Apply

- ☐ Burn it
- ☐ Bury it
- ☐ Put into the fields compost/manure
- ☐ re-use
- ☐ Sell to neighbors
- ☐ Throw it away
- ☐ Use as Livestock Bedding
- ☐ Use as Livestock Feed
- ☐ Other

Specify other.

---

190 How many drinkers do you currently use in MAIN broiler house?

____

191 Where do you get your water for your broilers? Please select all that apply

- ☐ borehole
- ☐ river/dam
- ☐ standpipe (treated water)
- ☐ well
- ☐ tap water
- ☐ Water vendors
- ☐ Other

Specify other.

---

192 Is the availability or quality of water a source of problem in raising your bird?

- ○ Yes
- ○ No

193 Do you USUALLY treat the water before putting into drinker?

- ○ No, I do not USUALLY treat
- ○ Yes, I boil
- ○ Yes, I treat with chemicals (disenfectant, chlorine)

194 How often do you clean the drinkers?

- ○ Never Clean
- ○ Twice a day
- ○ Once a day
- ○ 2-3 per week
- ○ Once or twice a month
- ○ Only after the production cycle

195 What do you USUALLY clean your drinkers with?

- ○ Water Only
- ○ Water and Soap Only
- ○ Water and disinfectant Only
- ○ Water, disinfectant, and Soap

196 How often do you USUALLY clean the feeders?

- ◯ Never Clean
- ◯ Daily
- ◯ Once or twice a week
- ◯ Once or twice a month
- ◯ Once a month
- ◯ Only after cycle ends

197 What do you USUALLY clean your feeders with?

- ◯ Water
- ◯ Water and Soap Only
- ◯ Water and disinfectant
- ◯ Water, disinfectant, and Soap

198 What feed distributor do you use? Please Select All that Apply

- ☐ NamFeed
- ☐ ZamFeed
- ☐ Novatec
- ☐ Tiger feeds
- ☐ Yielding Tree
- ☐ Olympia
- ☐ I use own mixed feed

199 Is feed stored on the ground or kept above the ground (for example, on a crate)

- ◯ above the ground (on crate)
- ◯ directly on the ground

200 Do you have a wheel bath or sprayers at the MAIN entrance to the farm where vehicles enter?

- ◯ Yes
- ◯ No

201 Do you have footbath(s)?

- ◯ Yes, I have footbaths at EVERY ENTRANCE
- ◯ Yes, but NOT at EVERY ENTRANCE
- ◯ No, I don't have ANY FOOTBATHS

202 What type are MOST of the footbath(s)?

- concrete trough with disenfectant
- concrete trough with water
- cut tyre of bucket with water
- cut tyre or bucket with disenfectant
- foot dip with disenfectant
- foot dip with water
- mat soaked with disenfectant
- mat soaked with water
- mattress soaked with disenfectant
- mattress soaked with water
- sisal sack with disenfectant
- sisal sack with water
- wood shavings soaked with disenfectant
- wood shavings soaked with water

203 How often do you clean the footbath (s)?

- Every day
- Once or twice per week
- 3 or 4 times per week
- Once a month
- Never change (just refills when it dries up)

204 Do you habe a hand bath?

- Yes
- No

205 Do you wash your hands between handling broilers at different houses

- Yes
- No

206 Do they have thermometers in any broiler houses?

- Yes
- No

206A: Do they have a heating source? If so, record

- ◯ No heating system
- ◯ Electric Heater
- ◯ Infra red lights
- ◯ Gas heater
- ◯ Charcoal
- ◯ Other

Specify other.

---

207 Protective Gear

208 Do you own any of these protective gear?	I do not have	I have	I use	I have for different houses
**209 Protective boots**	☐	☐	☐	☐
**210 Masks**	☐	☐	☐	☐
**211 overalls**	☐	☐	☐	☐
**212 Gloves**	☐	☐	☐	☐
**213 Goggles**	☐	☐	☐	☐
**214 Duster Coat**	☐	☐	☐	☐

216 How often do you clean the protective boots?

- ◯ after everytime entering the house
- ◯ in the evening
- ◯ after a few times in the poultry house
- ◯ only when dirty
- ◯ I don't have any protective gear

217 When going into the broiler house do people where protective boots/shoes/plastics?

- ◯ never
- ◯ rarely
- ◯ sometimes
- ◯ almost always

218 A Do you go into town wearing your gum-boots?

- ◯ Yes
- ◯ No

219 Do you USUALLY clean and disinfect housing and equipment after selling a batch?

- ◯ No
- ◯ Yes
- ◯ Not applicable, first batch

220 How long do you USUALLY let the broiler house rest before taking in the new batch?

- ◯ no rest
- ◯ less than one week
- ◯ between 1 and 2 weeks
- ◯ about 2 weeks
- ◯ about 3 weeks
- ◯ about 4 weeks
- ◯ Over a month
- ◯ Not applicable, first batch

221 Do family/friends/customers come and walk inside the broiler house?
For example, to pick birds to eat or to show off birds

- ◯ never
- ◯ rarely
- ◯ sometimes
- ◯ almost always

222 Do other animals go into broiler house?

- ◯ never
- ◯ rarely
- ◯ sometimes
- ◯ almost always

223 What animals Select ALL that Apply

- [ ] Cattle
- [ ] Dogs
- [ ] Ducks
- [ ] Geese
- [ ] Goats
- [ ] Guinea fowl
- [ ] Human House
- [ ] Local chickens
- [ ] No other animals/animal house
- [ ] Pig
- [ ] Rabbits
- [ ] Sheep
- [ ] Turkeys
- [ ] Other Broiler House
- [ ] Layer

224 FINALLY, A FEW LAST QUESTIONS

225 Does anyone at the farm have any training related to animal management?

- ○ Yes
- ○ No

226 What did the trainings cover? Please Select All that Apply

- [ ] record-keeping financial management marketing
- [ ] production
- [ ] animal health
- [ ] Other

Specify other.

___

227 Do you keep farm records? If yes, please list what types. PLEASE Select All that Apply

- [ ] I don't keep any records
- [ ] Mortality
- [ ] Feed Records
- [ ] Medical Records
- [ ] General Financial Records
- [ ] Other

Specify other.

---

230 What are your TOP sources of general news? That is news about weather, government, etc.

- [ ] Fliers / paper handouts
- [ ] Facebook
- [ ] Other Internet Sites
- [ ] People you know
- [ ] Print Newspapers
- [ ] Radio
- [ ] Television programs
- [ ] WhatsApp

231 Now, can I take a few pictures of your MAIN broiler house and briefly look inside?

232 Take photo of frontal view of the MAIN broiler house

If they say no, take picture of the ground

Click here to upload file. (< 5MB)

233 Record housing wall type of MAIN broiler house

- [ ] Iron Sheets
- [ ] Brick/Stone
- [ ] Wood/Timber
- [ ] Wire/Mesh
- [ ] Mud
- [ ] Other

Specify other.

---

234 Record housing floor type of MAIN broiler house

- ◯ A combination of wood and wire mesh
- ◯ Cement/Concrete
- ◯ Earthen
- ◯ Wire
- ◯ Wooden block (non-slated)
- ◯ Wooden slated floor
- ◯ Other

Specify other.

---

235 Record type of litter used in Main broiler house

- ◯ cow dung
- ◯ maize brans
- ◯ none
- ◯ rice husks
- ◯ sand
- ◯ saw dust
- ◯ Straw(Grass/Wheat)
- ◯ used paper
- ◯ wood shavings
- ◯ Other

Specify other.

---

236 Record drinker type (the majority of drinkers

- ◯ Commercial plastic drinkers
- ◯ Commercial metal drinkers
- ◯ Improvised (plastic)
- ◯ Improvised (metal)

237 Record if areas around drinkers are moist

- ◯ very damp
- ◯ somewhat damp
- ◯ dry
- ◯ Not applicable: Not Keeping Birds Currently

238 Record or ask how many feeders are in main broiler house?

____

239 Record feeder type

- ☐ Plastic
- ☐ Metal
- ☐ Wooden
- ☐ Rubber (tire)

241 Record the quality of ventilation in broiler house Select All that Apply

- ☐ 50 cm height of wall
- ☐ Gap in roof
- ☐ Have curtains on the open sides
- ☐ House height should be 2.6 minimum height and 2.4 on the back
- ☐ Structure is more than 10 meters away from other structures
- ☐ Two walls open along the length of the house not the width
- ☐ Broilers are kept inside the human house
- ☐ The broiler house does not have any of these

240 Observe the feed storage area. Is it damp?

- ◯ very damp
- ◯ somewhat damp
- ◯ dry
- ◯ Not applicable: Not Keeping Birds Currently

242 Record all animal houses that are within meters of the broiler house

- [ ] Cattle
- [ ] Dogs
- [ ] Ducks
- [ ] Geese
- [ ] Goats
- [ ] Guinea fowl
- [ ] Human House
- [ ] Local chickens
- [ ] No other animals/animal house
- [ ] Pig
- [ ] Rabbits
- [ ] Sheep
- [ ] Turkeys
- [ ] Other Broiler House
- [ ] Layer

243 Now, ASK THE RESPONDENT TO GO GET THE MEDICINE THEY USE

244 Record the MEDICINES they bring and them show them the posters and ask what medicines they NORMALLY use

- ☐ Akheri Bottle 200ml Green Bottle
- ☐ Akheri Powder 100 ml Green Bottle
- ☐ Akheri Powder 1l
- ☐ Akheri Powder 200 ml Yellow Bottle
- ☐ AKHERI Powder 500ml
- ☐ Albetong 25000mg
- ☐ Alfacycline 20 satchet
- ☐ Aliseryl WS Satchet
- ☐ Aminogrow WS satchet
- ☐ Amoxy-Col WSP Satchet
- ☐ Amprolium 20%
- ☐ Avitone atchet (Red)
- ☐ Bellmyo (Yellow Bottle)
- ☐ Boostermin
- ☐ Broiler Boot Multivitamin Satchet
- ☐ Buparvaquone 50 ml
- ☐ Chick Plus atchet
- ☐ CYDIP 1 liter
- ☐ Dexamethasome
- ☐ Diminafarm
- ☐ Doxin-200 ws
- ☐ endaam 200 ml (white bottle with cow)
- ☐ Enrocip 10% w/v
- ☐ Esb3 30% Satchet
- ☐ Flumesol-200 ws Satchet
- ☐ Gentadox ws Satchet
- ☐ Hi tet 200 LA Gold
- ☐ Hi-tet 120
- ☐ Interlocy Bottle
- ☐ IRON 100 INJ
- ☐ Ivertong 1% 100mjl
- ☐ Ivertong 1% 15ml
- ☐

- [ ] Ivertong 1% 50ml
- [ ] Ivertong 25ml
- [ ] Ivervet
- [ ] Leva-200 WS
- [ ] Limoxin 25 Spray
- [ ] Megapen LA
- [ ] Multivitamin 100ml
- [ ] Multivitmain 50ml
- [ ] Oxyfarm (white bottle with red cap)
- [ ] Oxyfarm 20%
- [ ] Oxytetracycline 50% WSP Satchet
- [ ] Parvexon 100 ml
- [ ] Phenix Sresspac satchet
- [ ] Piperate 100g satchet
- [ ] Piperin WS Satchet
- [ ] Poultry tonic 1 liter
- [ ] Poultry tonic 500ml
- [ ] Pro-Pen
- [ ] Sulfa 333
- [ ] Sulfadimidin WS
- [ ] Sulfadimine Sodium 100g
- [ ] SUPA KUKU Premix Bag
- [ ] Tetrocy Egg Formula Satcht
- [ ] Tetrocy LA
- [ ] Tetroocy Chick Formula Satchet
- [ ] Tickle
- [ ] Ultracide 100ml
- [ ] Ultracide 500 ml
- [ ] Vet Oxy 33%
- [ ] Viruguard 100ml
- [ ] Virukill 500 ml
- [ ] Virukill 1 liter
- [ ] Virukill 100 ml
- [ ] Vitamin E Stress Pack

- [ ] Vitamin Stress Pack
- [ ] Zundu Natural Booster Big Bottle
- [ ] Zunfu Natural Booster small bottle
- [ ] Other

Specify other.

---

245 We would like to collect fecal samples at a later date. We would collect fecal samples from poultry, swabs, and water. We will follow best protocol for entering the poultry house. Would it be okay if we came back?

- ◯ Yes
- ◯ No

closing

Tell the informant that you appreciate their time and that their responses will help us succeed in our project.

Also tell them that after analyzing the results we will come and talk to them about what we found and get their inout on interventions to improve health of their birds.

REMEMBER to write their last name and ID on next page. For example Caudell_100 ALSO REMEMBER TO DO THE ID SURVEY
