# Supplementary material for: Towards a bottom-up understanding of antimicrobial use and resistance on the farm: A knowledge, attitudes, and practices survey across livestock systems in five African countries
Source: PLoS One. 2020 Jan 24;15(1):e0220274. doi: 10.1371/journal.pone.0220274 (PMC6980545; doi:10.1371/journal.pone.0220274)
Supplement: Attachment — Submitted filename: Letter to Editor PONE-D-19-19328.pdf [file pone.0220274.s004.pdf]

THE OHIO STATE UNIVERSITY

Leah E. M. Bevis

Assistant Professor, Department of Agricultural,
Environmental & Development Economics

329 Agricultural Administration Building
2120 Fyffe Road, Columbus, OH 43210

614-292-4749 (Phone), 614-292-4749 (Fax)

August 24, 2019

Dear Dr. Yildirim,

This manuscript claims to (and I think does) provide the first survey data on antimicrobial use on African farmers. It summarizes survey data on antimicrobial use and on knowledge about correct antimicrobial practices from locations within 5 African countries. Each of these locations represents a different sort of poultry (and more generally livestock) production system. I think this is a valuable dataset, and on the whole a well written paper, presenting novel evidence on a subject that will likely become more and more important over time.

To my mind, there are two primary flaws in the paper, each of which can be addressed to some extent. First, the authors provide very little information on their sampling strategies --- it is therefore unclear whether their results can be taken as representative for any geographic area or community. Likely, the answer is that results are not statistically representative for any given population. But the authors need to make this clear, and ideally to give us some information on whether their samples can be taken as "indicative," if not statistically representative, of any larger population. This would help us to think about antimicrobial use in Africa more generally.

Second, the analysis could be more clear in two ways. First, they could simply write about the analysis more clearly. There are so many statistics that one gets a bit lost, and I felt that key highlights were not clear. Second, the authors use ordinary least squares regression to predict 3 index variables representing "knowledge," "attitudes" and "practices" --- but it is not clear what those three index variables really mean. That is, as a reader, I have no idea what a high vs. low mark for "attitude" means, after looking at those attitude questions. So at the moment, the regression results are basically meaningless.

I think if these issues could be cleared up, the paper would be quite valuable.

Sincerely,

Leah Bevis

Leah E. M. Bevis
