# Supplementary material for: Towards a bottom-up understanding of antimicrobial use and resistance on the farm: A knowledge, attitudes, and practices survey across livestock systems in five African countries
Source: PLoS One. 2020 Jan 24;15(1):e0220274. doi: 10.1371/journal.pone.0220274 (PMC6980545; doi:10.1371/journal.pone.0220274)
Supplement: Attachment — Submitted filename: Reviews PONE-D-19-19328.docx [file pone.0220274.s005.docx]

Dear Authors,

I think this is a valuable manuscript with important implications. Yours appears to be the first, or at least one of the first, studies of antimicrobial drug application to livestock in Africa. What follows are a few comments that might improve the manuscript further. My primary concerns are in bold, and I will summarize them for you here too: **(1)** It is unclear whether your results can be taken as representative for any geographic area or community. Probably not. If not, can the samples can be taken as “indicative,” if not statistically representative, of any larger population? This would make your results more valuable. **(2)** It is not clear what the three index variables (“knowledge,” “attitudes” and “practices” ) mean. As a reader, I have no idea what a high vs. low mark for “attitude” or for “practices” means, after looking at the attitude and practices questions. So at the moment, those regression results seem fairly meaningless.

1. You introduce your study locations by saying that 3 production systems were targeted, which makes sense to me. (You could be a bit clear though about the poultry type --- I assume broilers and layers are only chickens? And pastoralists are primarily cows?) However, much more info is needed:

A. **You do not mention how you chose the specific locations w/in the country.** In fact, it’s not even clear in the main manuscript that you are surveying only in specific places --- somebody skimming quickly might think these were country-wide surveys. You need to justify your choice of location(s) for each production system (i.e., Lusaka Province and Central Province in Zambia and the 6 districs in Zimbabwe for broilers, Dormaa Central Municipal of Brong Ahafo Region in Ghana and Gatundu North Subcounty in Kenya for layers, Longido District in northern Tanzania for pastoralists). In some cases these choices are justified in the appendix, but not always.
B. **Equally or more important, you need to mention your sampling strategy, within each location chosen**. You claim that you discuss sampling in the appendix, but you do not do so for Ghana or Kenya. You mention it briefly for Tanzania but in not enough detail -- for instance, you say that you “target” adults at the market, but how? Did you have a list? Did you walk around? How did you sample them? Or did you attempt to survey *all* of them (which would be a census, not a sample, for that particular market)? Similar questions regarding the household interview --- how did you find the households? How you did you sample, once you had a list of some sort? You are not even attempting to describing your sampling techniques in anything close to a formal manner, and to my mind, this really damages the credibility of the paper and the results. Only for Zambia and Zimbabwe do you mention your sampling frame and your sampling strategy, and for Zimbabwe more detail is needed on how the snowball sampling was performed.
C. **You give no information on power calculations**. This, of course is related. For instance, you decided to go with 200 people total in the 4 Zambaian camps. How did you choose that number? Ideally you would have done power calculations beforehand, to be sure that you could find meaningful statistics at the chosen sample size. If you did not do that, explain how you *did* pick those sizes.
D. **Hand in hand with those questions is the question of “representativeness”** -- if I survey randomly within a certain district, my results may be said to be representative at the district level. It is not clear whether your results are representative for any market, district, etc. If not, that’s unfortunate, but it doesn’t kill your paper. However, you need to be clear.
E. **Who is being surveyed, in each household?** How do you choose your respondents? This is also commonly reported information for any survey.

2. You need to be more clear about livestock included / not included in the survey. Are all livestock being represented or only birds? I think it might be only birds, as those are the only ones you speak about, but you never explicitly state it. This is particularly confusing for the Maasai since most of us will first think of cattle, w/ the Maasai.

3. You say that you chose those 3 production systems (layers, broilers, pastoralists) according to several listed factors. But the next sentence is not clear -- you list the concept but not the goal. That is, you chose according to what objective, for those variables? You wanted to choose production systems that made a significant contribution to GDP? (Presumably.) That were projected to grow significantly in future? And what about the patterns of AMU? What sort of pattern were you looking for? You should make it clear what your objectives were, when choosing these production systems. You should also explain why you chose those production systems in THOSE countries. Why Zambia, not Uganda? Why pastoralists in Tanzania rather than Ethiopia?

4. Where the KAP surveys identical across countries/production systems? If so, why would you do this? (Surely, for instance, some different questions are relevant to pastoralists than to folks owning chickens.) If not, how were they different across contexts?

5. I do not understand why you are summarizing figures/statistics by country, rather than by production system, in the results. You chose your locations by production system. And your results are far from representative for any given country. So statements like “Ghana(ian) farmers kept the largest flocks” or “Kenya(n) farms had a median of 700 birds” seems misleading to me. First of all, you cannot say anything about “Ghanaian farmers” or “Kenyan farmers” in general. Second of all, isn’t the whole point to examine different production systems? So if anything, why not talk about farms keeping broilers vs. farms keeping layers vs. pastoralist farms? Are systems similar between Kenya and Ghana, given that you were targeting farmers keeping layers? It looks like not, from your results.

6. I do not understand why you are pooling all of your data, in analysis, across countries and/or production systems. Relationships might differ significantly across contexts, so it seem imperative that you first present disaggregated analysis before pooling data.

7. Ordinary least squares should not technically be applied to a count (rather than continuous) outcome variable. It is likely that using a more appropriate model will not make any difference, but you should still switch to a model that is appropriate for count data. Many websites and textbooks survey such models, e.g.: <https://stats.idre.ucla.edu/stata/seminars/regression-models-with-count-data/>

8. **More importantly, it is not clear what is learned by these “total” K, A, P variables that you use in regression.** Knowledge is perhaps understandable --- it would seem that the higher this count variable is, the more knowledgeable the farmer is about antimicrobial medicines. But the Attitudes questions are not clear --- what it the “right” answer to each of these questions? If you have a high vs. low score, what does this say about your “attitude” towards antimicrobial medicine? If you cannot clearly state that a high score means this, and a low score means that, then it is not appropriate to use this variable as an outcome variable in a regression. Precisely the same point applies to practices -- I have no idea, from those questions, what it means if a farmer has a higher or lower practice count.

**If you cannot give an intuitive meaning behind those totals, then you should not use them.** Simply summarizing your survey information, by question, across contexts, is fine. It would be better not to use an index variable (the K, A, P totals) if it is not meaningful.

9. Your results and discussion could be better organized. The findings and statistics are all interesting, but it’s not always clear what holds each paragraph together, and in general I find it hard to read these sections and keep in mind the “highlights” or the most important findings. It feels too much like a list.

Very minor comments

- Personally, I could do with many fewer acronyms. For instance, I do not see the point of using HMIC and HIC, and I think the use of KAP really hurts the readability of the paper. Even AMU vs AMR is confusing. This paper would be much easier to read if the number of acronyms was reduced.
- Thematic analysis of WHAT qualitative data was used to develop the KAP survey? The source of qualitative data, and which countries and production systems were represented in this qualitative data, should be stated in the main manuscript.
- Similarly, where were the survey pilots conducted? In each location? You need to be more specific about such details; the manuscript has many such details that are lacking.
- You have a “Figure XXX” in the supplementary material on locations, under Tanzania.
