# Supplementary material for: Towards a bottom-up understanding of antimicrobial use and resistance on the farm: A knowledge, attitudes, and practices survey across livestock systems in five African countries
Source: PLoS One. 2020 Jan 24;15(1):e0220274. doi: 10.1371/journal.pone.0220274 (PMC6980545; doi:10.1371/journal.pone.0220274)
Supplement: Attachment — Submitted filename: Response to Reviewers.docx [file pone.0220274.s006.docx]

| **Editorial** | |
| --- | --- |
| We note that you have reported significance probabilities of 0 or <0 in places. Since p=0 is not strictly possible, please correct this to a more appropriate limit, eg 'p<0.0001' | Thank you, all instances have been changed |
| In your Methods section, please provide additional location information, including geographic coordinates for the data set if available. | We have provided maps with the approximate locations of the study area. Given that we discuss sensitive information (views of farmers on local veterinarians) we do not want to publish latitude/longitude data. |
| Please provide additional details regarding participant consent. In the ethics statement in the Methods and online submission information, please ensure that you have specified whether consent was suitably informed. | We have provided additional details of consent in Lines 234-236. |
| We note that Figures 2 and Supporting Information Figures]in your submission contain map/satellite images which may be copyrighted. We require you to either (1) present written permission from the copyright holder to publish these figures specifically under the CC BY 4.0 license, or (2) remove the figures from your submission | We have removed all copyrighted figures from the manuscript and supplement and replaced with figures that comply with the CC BY 4.0 license and cite sources. |
| Please include captions for your Supporting Information files at the end of your manuscript, and update any in-text citations to match accordingly. | Captions have been provided for Supporting Information files and in-text citations have been updated accordingly. |
| **Reviewer 1** | |
| Comments | Response |
| The authors made a good attempt to evaluate the knowledge, attitudes, and practices related to antimicrobial use and resistance in livestock farmers. The paper is interesting and well written. However, there are some suggestions for its improvement. In Introduction, a separate literature review section may be added. The discussion lacks strong support from previous studies which should be checked. | We agree that our review of the literature could be expanded and have done so in Lines 106-140. In addition, we have made it clearer that there is limited literature on knowledge, attitudes, and practices related to AMU/AMR in these areas and production systems and therefore contextualizing our results with previous studies is limited by the lack of similar studies. Notably, several studies have come out since we originally submitted the paper and we have added these into the Discussion on Lines 698-700 |
| **Reviewer 2** | |
| **Comments** | **Response** |
| Abstract: The abbreviation AMR is used in the abstract but is not defined. | Thank you, we have removed the abbreviation from the abstract |
| There are a lot of abbreviations used in this paper. In my opinion this is very distracting. I am fairly sure the full version of AMU is never given. Please limit the use of abbreviations, and make sure all remaining abbreviations are defined at least once. | Thank you, we have limited the use of abbreviations to those most commonly used in the literature (i.e., AMR, LMICs, and KAP) and have defined abbreviations used. |
| Ethnographic approach was including in the abstract but not addressed in the survey development. It is again discussed as a limitation. The definition of this approach and how the distribution of this survey either meets or does not meet this approach is needed. | Thank you, we have clarified how our study relied upon an ethnographic approach to this work in Lines 200-203 |
| More information regarding the types of questions included in the survey instrument are needed. Questions are discussed in the analysis, but the general outline of the survey instrument is not provided in the materials and methods. | We agree that more information should be presented on the actual questions, particularly for the questions comprising the KAP questionnaire. To this end, we have modified the Table covering the KAP questions (Table 2) and made it clearer in the preceding section how questions were asked and in Lines 216-223 |
| More information regarding the methodologies used are needed. The name of tests and methods are often given without citations, explanations for use, or interpretations are given. | We agree that the names of tests/methods could be further interpreted for the reader, therefore provided more information on these methods within the text (see specific comments below). |
| It seems that the term antimicrobial and antibiotic are used somewhat interchangeably in this paper. I do not think these two terms are equivalent, definitions are needed to point out the differences if you are using them intentionally. | We have changed all references to ‘antibiotics’ to ‘antimicrobials’, unless we are referring to a specific antibiotic drug and explain the reason behind the word change. |
| It seems odd to describe the variables used in models as bullet points below a table | We agree placement of definitions below tables is awkward. In the revised manuscript, we have included a new “Variable Definition Table” (see Table 3) in the Methods and then direct readers to this table in the Results section. |
| The models should be better defined in the materials and method section. I could not follow the tables. I wasn’t sure which results were from the OLS models, etc. The models should be clearly written out with all variables explained in the materials and methods section. | We have provided more details on our modeling approach and in the caption of the Results tables. We have also included a new variable definition table (see Table 3) |
| Are you sure OLS is the right approach? It looks like to me you ran three models: knowledge, attitude, practices. Is it possible that these models have correlated error terms? In this case a seemingly unrelated regression would be more appropriate. This could be incorrect, but as the models are not defined right now it is unclear to me that OLS is the correct model. | We thank the reviewer for the suggestion of using seemingly unrelated regression in place of OLS. We agree that correlated error terms could exist and so reran all models using seemingly unrelated regression. Results showed that relationships documented in OLS between dependent and independent variables remained similar in magnitude and significance. Given the interpretation of OLS (as a modeling method) is easier to convey, we retain the OLS results in the paper. We have attached the outputs of the Seemingly Unrelated models to the revisions (title Seemingly Unrelated Regression) |
| Materials and Methods: What is Kobo Collect? A software program that hosted the survey instrument? Please define. | We have clarified what Kobo Collect is on Lines 225-226 |
| Please define and explain how the survey approach met the requirements of a purposive sampling approach. | This has been further explained in Lines 245-247 |
| Tukey-Kramer comparison- This method needs further explanation and a citation. How are the results interpreted? | -We have added a citation and further explanation in the Methods section (Lines 285-287) and Results, (Lines 457-460) |
| Table 1: From my understanding the recoded values is used as an indicator of the “right” answer. For the statement “Giving animals antimicrobials can help them GROW BIGGER…” answering disagree was coded with a 1. If the animal was sick giving antimicrobials would potential result in these positive outcomes. Additionally, there is evidence of increased production with the use of sub therapeutic doses of antimicrobials, I understand this is not a “good” practice, but if we are simply considering correct vs incorrect the statement is actually correct. I have a similar issue with the statement “Using vaccines can reduce use of antibiotics.” Vaccines can be for viral (more common) or antimicrobial issues. So depending on which vaccine the respondent was thinking of, or more familiar with the “right” answer changes. | The reviewer has a good point and brings up a discussion we have had with coauthors. Our assignments of “right” attitudes, should really be interpreted as “recommended”, “prudent”, or desirable based upon what we know drives antimicrobial resistance. We have made this qualification more important in Lines 274-280 and in a new table defining KAP scales (Table 1). In addition, in our question for animal growth, we specified that giving “healthy” animals antimicrobials can help them grow bigger…”. We have updated the table to reflect this important addition (Table 2). |
| Cook’s D- this method needs a citation as well as an explanation. | A citation had been provided and an explanation of the measure and how the measure was used to perform analysis has been provided (Lines 318-320) |
| These controls were not collinear with variables…” how was this determined?” | We assessed multicollinearity through calculation of variance inflation factors for each model. We have made this clearer in Lines 322-324. |
| Shapiro-Wilk Test- citation is needed? What is the interpretation of this test? | A citation has been provided and interpretation has been further expanded in Line 328. |
| Antimicrobial knowledge and Use Patterns: How is a treatment failure defined? | Treatment failure was a respondent-centered definition, in that we asked the respondent whether they noticed an increase in antimicrobial drugs not working to treat disease. We have clarified this definition in a “Variable Definition” Table 3 |
| KAP and on-farm dynamics Table 4 is mentioned in the text but is missing. I think table 5 should be re-labeled. | We believe the reviewer meant Table 5 was listed but missing. We thank them for noticing this and have updated the Table numbers accordingly. |
| What is meant by correlates in Table 6? What method was used to obtain these results? | We thank the reviewer for catching this. We have changed “Correlates” to “Associations between” to maintain consistency with other tables. |
| Check for missing commas throughout to improve understanding. | We have added commas to improve readability. |
| Pg 5 line 117 missing period after [8,9] | A period has been added. |
| **Reviewer 3** | |
| Comments | Response |
| The authors do a good job motivating the need to understand the factors behind the misuse and possible abuse of antimicrobial drugs in LMI countries, but it was not clear why they decided to focus on these specific 5 countries (Ghana, Kenya, Tanzania, Zambia and Zimbabwe) and livestock systems. I would like to see a detailed explanation of why they chose those specific countries/systems in Africa, and why not also other LMICs in Asia or Latin America. | The project was funded by the Fleming Fund of the United Kingdom, which set out a series of criteria with which FAO and government partners considered in their selection process. While the project also includes countries in Asia, the FAO teams there started earlier and used different instruments, thereby limiting harmonization with our results. We have included a more detailed explanation of country selection in the manuscript in Lines 159-187. |
| Within each country-livestock system, the choice of regions in the sampling frame was well justified (namely, regions where that livestock system was most prevalent in the country). However, the sampling strategy felt disjointed, as if the 5 projects were independent and glued together in the last minute. For example, in the description of study locations and sampling in the supplement, much more detail (and additional information) is offered for Tanzania and Zimbabwe than for other countries. | While we acknowledge differences in sampling approaches due to “census records, reachability of households, financial resources, and time constraints” we do agree that more detail is needed for each country. To this end, we have added a “Sampling Procedure” for each country in the Supplement (S1) and have provided more detailed information on KAP development and deployment. |
| More fundamentally, I am concerned about the comparability and “poolability” of results. While the regression analysis addresses some of this concern by including country dummies, I wonder if there are also country-specific associations with some of the independent variables included in the regression (e.g. the coefficient on the variable farm scale, or education, or agrovet advice could be country specific). To alleviate this concern this, in addition to splitting the sample between pastolarists and everybody else, I would like to see the results of the regressions for each individual country. Perhaps they are very similar and can be pooled, but it would be good to check if that is the case. The same apply for the descriptive statistics. While for the sake of conciseness it may make sense to report descriptive stats for the whole sample, if there were marked differences across countries that wouldn’t be advisable. Those differences should be highlighted. | Our major concern in the analysis was looking at the patterns of AMU/AMR KAP and correlates across production systems. We include country as a control (in the case of poultry farmers) to assess those factors consistently related to AMU/AMR KAP. Nevertheless, we have run regression models separately for each country for those interested in country relationships and have added these models to the revision file.  We also agree with the reviewer that descriptive statistics should be provided by country. We provide these by-country statistics in the Descriptive Results section Lines 369-448. In addition, we have added country-specific descriptive statistics for sources of health advice and the persons administering drugs, withdrawal, prescription practices, and KAP components in the Supplement (S1 Appendix). |
| The key dependent variables, knowledge, attitudes and practices are each a weighted average of a number of underlying variables. While I can see the value in doing this, I also wonder about explaining some of the individual components that may be particularly important for the fight against antimicrobial misuse – for example, if giving animals antimicrobials to make them fatter in Tanzania is identified as a key misconception, what are the factors explaining this? | We agree with the reviewer that regressions examining specific components of the KAP (e.g., prophylactic use) would prove useful in identifying the specific practices promoting selection for AMR emergence and spread. Indeed, we intend to carry out these analyses with FAO country teams in the next stage of the project. The purpose of the analysis will be to inform the development of targeted interventions within countries. For the current analyses, we wanted to provide a cross-cultural examination of the factors related to AMU/AMR knowledge attitudes and practices. |
| There are many typos in the presentation of the descriptive results; please revise the numbers carefully. Some of the ones I’ve caught: - Line 288: should be 48 (not 41) percent. - Line 289: 22 (instead of 21) percent. - Line 290: Zimbabwe instead of Zambia. - Line 301: Maasai households average 9 persons - Line 302: Ninety-three percent “of respondents” (not “of farm owners”) – since not all respondents are farm owners. - Line 304: Swap Kenya and Zimbabwe. - In Table S2 and S3 there are some inconsistencies. The frequencies in Table S3 do not add up to those in Table S2. - Line 343: 65 (not 63) per cent | We thank the reviewer for noticing these errors, these corrections have been made. |
| The education variable is treated as a linear variable. However, moving from a 0 (none) to 1 (primary), is not the same than moving from a 1 to a 2 (secondary). I would recommend including education as a set of dummies for primary, secondary, and tertiary and above, where “none” is the reference category. | We thank the reviewer for this suggestion and agree. We have entered education as dummy variables in the new models. |
| The authors estimate three different regression models to explain knowledge, attitudes and practices regarding antimicrobial use and resistance; each with a different set of regressors: demographics, farm characteristics and antimicrobial use, and sources of animal health advice. Why not estimate a single, comprehensive model including all the explanatory variables? This would help alleviate the risk of omitted variable bias, especially if some of the regressors are correlated. | We again thank the reviewer for a helpful modeling suggestion. We have added combined models in the manuscript that include all significant variables in earlier models. Given the large number of variables, we use backward selection at the 0.05 significance level for retaining variables in the final model |
| In the regression models, some of the independent variables are arguably caused by the variables they are meant to explain. I am thinking specifically about treatment failure (which is also one of the most important implications of microbial misuse). The finding that around 50% reported to observe an increase in treatment failures is interesting and important by itself, and worth being further explored – perhaps in a regression analysis. | We agree with the reviewer that the path of causation may sometimes proceed from an outcome variable (i.e., knowledge of AMR) to independent variable (e.g., treatment failure). Due to this, we are careful not to suggest we are looking at causation but instead emphasize association. For example, the captions of our results tables begin with “Associations between”. However, we do take the point that in order to develop interventions, determining the direction of causation is critical. To this end, we have added a sentence in our limitations section calling for longitudinal research (Lines 750-753) |
| Regarding the policy implications of some of your results: In some countries, antimicrobials are cheaper when purchased with a prescription. Is this the case in the countries you are analyzing? The fact that only 12% of the households use prescriptions regularly suggest that’s not the case, or that the price differential does not justify seeking a prescription. Could you please elaborate on the merits of such policy (and others that are being used in high-income countries) in your context? | We thank the reviewer for bringing this to our attention. We are aware that drug subsidies are common specially for human use, but the situation is different in LMICs. Although we have not conducted a legal assessment in project countries, we do suspect that subsidies are not available for use of antimicrobials in animals. We do recognize, however, the more general argument revolving around the importance of making the “economic argument” in addressing AMR so have added discussion of economic implications throughout the Discussion (e.g., Lines 701-705) |
| From the questionnaire, it is not clear if the limited access to animal health professional is by choice or it is a constraint. Questions 83 and 85 only ask about where respondents get information/advice, and how often certain groups people give them advice, but are mute as to whether they face challenges getting advice from their favorite source. Question 100 captures these challenges more directly and I would recommend that the authors report the statistics of this variable more prominently. | From our qualitative work, our view is that the lack of veterinarians is the initial constraint that ultimately drives future choices not to consult a vet (see Figure 1 in the main manuscript). Farmers would recall trying to call the veterinarians, but they were busy or far away. This combined with ideas that “they always give us the same medicine anyway” leads farmers to bypass the vet and instead head directly to agrovets. We agree that specifics of these challenges are important and so have reported statistics in the paper in Lines 443-445. |
| I do not think that results reported in the paper “stress that intervention philosophies based primarily upon top-down approaches are unlikely to result in broad changes in AMU within LMICs” lines 531-533. The paper simply doesn’t ask this question. (Moreover, at other points in the paper the authors point at the negative impact of the structural adjustment policies that reduced public veterinary services on AMU– They cannot have it both ways). | We agree with the reviewer that our results do not support the notion that top-down intervention philosophies are unlikely to result in broad changes. We have toned down this language in the Discussion. Instead, we build off previous research showing the impacts of structural adjustment on regulatory and veterinary health capacities, then highlight that this research empathizes community-targeted interventions, and then suggest our results provide insight into these interventions |
| Regarding interventions aimed at agrovets (line 541 on), another factor to consider is the possible lack of “incentive compatibility”, that is, as agrovets profit from the sale of AMs, they may have an incentive to recommend overuse. | We thank the reviewer for this point. To address this point, we have included discussion of how the relationship between the agrovet-farmer (i.e., a principal agent relationship) can give rise to a moral hazard in respect to drug sales (e.g., Lines 701-705) |
| I do not think that your results emphasize the importance of bottom-up strategies to address AMU and AMR (lines 569-570) – The study documents dimensions of knowledge, attitudes and practices related to AMU and AMR in livestock systems, and identifies the livelihood factors associated with these dimensions (lines 54-56). Sure, all of this is important to inform bottom up interventions, but claiming that your results support those bottom up strategies is going too far (i.e.,will be more effective) . | We agree with the reviewer that our results, considered by themselves, do not support the notion that bottom-up strategies will be *successful* in addressing AMU/AMR and we have edited any language suggesting otherwise in the Discussion. We do believe, however, that our results emphasize the importance of bottom-up strategies to address AMU and AMR considered in the context of the project countries, given that farmers have limited interaction with stakeholders who can be regulated from the top-down (veterinarians, laboratories) and by demonstrating high rates of self-administration and treatment and a lack of prescriptions (another indicator of weak regulatory capacities). |
| Line 109 and 111: Please define One Health more explicitly. | We provide a more explicit definition on Lines 63-66, |
| - Lines 191-192: would this include veterinarians as a separate category from district veterinary officers and community animal health workers? | Veterinarians and district veterinary officers would be considered under the same title. In our study, veterinarians are those with DVMs. We have provided a broader categorization using “public and private” veterinarians instead of district veterinarians to prevent confusion. |
| Line 263: should be “withot” not “with” | This had been changed. |
| Line 298: Spell out CRD. I would also recommend give a brief explanation of what these diseases entail. | We have spelled out CRD. Due to length considerations, however, we direct the reviewer to sources that explain these diseases, which are common in poultry, within the Supplement in Table 7. |
| Table S4: The variable Read does not belong with the type of livestock, I would move it to the section that includes education level variables. | We thank the reviewer for the suggestion. We have updated the table. |
| Line 327-328: present these variables in tables of descriptive statistics. | These statistics have been added to a new table in the appendix. Table 3 in the Supplement. |
| Lines 356: I do not know how to interpret this figure. Is it supposed to be a pdf or a histogram? Can you please explain? | The figure is a distribution of the correct responses for knowledge, attitudes, and practices. We used connected lines because showing the distributions with histograms for every country, in the same figure, was complicated. That is, the histograms were overlapping and confusing. We have provided a clearer description of the figure on Lines 466-467. |
| **Reviewer 4** | |
| **Comments** | **Response** |
| • In the AMR abstract please specify …………… (line 72) | Thank you, we have removed the acronym from the abstract. |
| • At the end of the introduction, please state the objective of this study | We have added a sentence detailing the objectives of the study at the end of the Introduction in Lines 150-152. |
| In the method of writing a more specific regression model for example Y = f (X1, X2, ...) so that it can explain the model in Tables 3, 4, 6. | We have included more information on the specific regression models in the Methods in Lines 294-309 |
| In the method not mentioned Pearson's Correlation analysis tool, the results suddenly appear in Table 2 | We thank the reviewer for noticing this omission. We have mentioned the Pearson’s correlation in the preceding paragraph. |
| In the "results" Table 5 ...? | We thank the reviewer for noticing this omission, we have updated the table numbering. |
| **Reviewer 5** | |
| **Comments** | **Response** |
| The outputs of the focus groups discussion and Key informant Interviews should be presented in the paper | We agree that more information could be provided on the themes emerging from analysis from the qualitative data and have provided an overview in Lines 336-368. In this section, however, we only focus on the major cross-cutting themes identified. We are currently in the process of writing up a manuscript that goes over the qualitative results in greater detail. |
| Literature review on the topic needs to be presented | We agree that our review of the literature could be expanded and have done so in Lines 106-139. In addition, we have made it clearer that there is very little literature on knowledge, attitudes, and practices related to AMU/AMR in these areas and production systems. |
| The developed conceptual model is not based on theories or empirical work | We agree we take an inductive approach to examining AMU/AMR and related practices across the five project countries. Given there is little theoretical/empirical work on AMU/AMR KAP across these contexts we do not have a theoretical perspective to draw upon, although we do use empirical work to inform development of our survey. This has been further outlined in the Literature Review (Lines 106-139). |
| What is the difference between KAP measures and constructs? did you carry out factorial analysis where is the output of the analysis? | We thank the reviewer for pointing out this word usage. The KAP measures represent a linear scale of responses and so should properly be referred to as scales or measures. We have removed all reference to “constructs” |
| The output of the OLS analysis is not presented. | For the OLS results, we report the Betas, 95% CIs, R-squared, and observation numbers. We realize that more statistics are available and are happy to include more statistics if the reviewer provides suggestions. |
| Why re-coding of the scales is necessary since it has decreased information? | We recoded the KAP scales so we could combine the scales into a continuous outcome in an OLS model. That is, each KAP measures could take on a range of values from 0%-100%. |
| the paper requires a lot of improvements in terms of methodology and analysis | We hope we have improved the methods and analysis through addressing the reviewer’s comments and suggestions above. |
| **Reviewer 6** | |
| **Comments** | **Response** |
| The major results of the research is the level of knowledge, attitude, and practice (KAP) across producers in Africa. Could the manuscript be improved by including tables of sample means for the variables defined in table 1 for each country? Most of the important results, discussion, and implications have to do with these summary statistics... the regressions do add information, but the sample means are important to understanding what is going on. | We agree that country-specific information on KAP scores would improve the manuscript. We provide these in the Supplement (Table 15) given considerations of manuscript length. We do provide country-specific KAP composite scores in the manuscript to highlight differences in composite means across countries. |
| The comparison across practices (layers, broilers, and cattle) may confound the regression results. The reasons for using antimicrobials are much different across practices (Figure 3), the dosages are much different... a table of means would help here, as would adding a variable for practice type in the regressions... partly captured by country variables, but may want to add practice variables. | Thank you, we agree that comparisons of practices across production may confound results and have separated analyses into poultry and pastoralist systems. We group poultry systems as interaction effects between specific poultry systems (broilers, layers) and correlates of AMU/AMR KAP were not significant. In the Supplement, we now provide a tables with means for all variables in our models (**we bold these variables**). |
| Is there a conflict of interest between the antimicrobial supplier and the producers? Is it in the best interest of the suppliers to maximize sales? How does this affect KAP scores? How does that affect education efforts? | We thank the reviewer for this point. To address this point, we have included discussion of how the relationship between the agrovet-farmer (i.e., a principal agent relationship) can give rise to a moral hazard in respect to drug sales. We include these issues throughout the Discussion (e.g., Lines 701-705) |
| (4)These results indicate that producers in five African nations might use "too much" input... in other research, African producers may not use "enough" medicine... (eg. Marsh, Foot and Mouth Disease). Is there an explanation between these types of results? | This is an interesting question and one that may, by identifying the correlates of “too much” or “too little” across productions systems be important for interventions to limit AMR. In this work, while we did collect general indicators of antimicrobial use, we did not collect the type of detailed data to determine whether the farmers were overusing antimicrobials (diseases present, dosages given per animal) so cannot look at correlates of “too much”. However, we do plan on examining specific practices across these production systems in later analyses (“who is likely to report not finishing the dose), which we hope will provide some answers to this important question. |
| **Reviewer 7** | |
| **Comment** | **Response** |
| This is an interesting and important topic. The manuscript is well written and for the most part easy for the reader to follow. There are two very important problems to be fixed. First, the Education variable is used in the regression analysis as though it was a continuous variable - IT IS NOT A CONTINUOUS VARIABLE - thus the regression is invalid and incorrect. The education variable needs to be included in the regression as 3 dummy variables. | We thank the reviewer for pointing out this modeling error. We have entered education as a continuous variable in the new models. |
| Second, the graphs are difficult for the reader to follow and labelling (titles and labels on axes) are insufficient | We have improved all titles/labels on all figures. |
| **Reviewer 8** | |
| **Comments** | **Responses** |
| I think this is a valuable manuscript with important implications. Yours appears to be the first, or at least one of the first, studies of antimicrobial drug application to livestock in Africa. What follows are a few comments that might improve the manuscript further. My primary concerns are in bold, and I will summarize them for you here too: (1) It is unclear whether your results can be taken as representative for any geographic area or community. Probably not. If not, can the samples can be taken as “indicative,” if not statistically representative, of any larger population? This would make your results more valuable. (2) It is not clear what the three index variables (“knowledge,” “attitudes” and “practices” ) mean. As a reader, I have no idea what a high vs. low mark for “attitude” or for “practices” means, after looking at the attitude and practices questions. So at the moment, those regression results seem fairly meaningless. |  |
| **You do not mention how you chose the specific locations w/in the country.** In fact, it’s not even clear in the main manuscript that you are surveying only in specific places --- somebody skimming quickly might think these were country-wide surveys. You need to justify your choice of location(s) for each production system (i.e., Lusaka Province and Central Province in Zambia and the 6 districs in Zimbabwe for broilers, Dormaa Central Municipal of Brong Ahafo Region in Ghana and Gatundu North Subcounty in Kenya for layers, Longido District in northern Tanzania for pastoralists). In some cases these choices are justified in the appendix, but not always. | We agree with the reviewer that more information is needed on the sampling of project countries and the sampling decisions within countries. We have included these details in Lines 159-187 and provided more detailed reasons behind within-country sampling and production system selection in the Supplement. In brief, potential countries were selected by the UK government and finalized with discussions with FAO/government partners. |
| **Equally or more important, you need to mention your sampling strategy, within each location chosen**. You claim that you discuss sampling in the appendix, but you do not do so for Ghana or Kenya. You mention it briefly for Tanzania but in not enough detail -- for instance, you say that you “target” adults at the market, but how? Did you have a list? Did you walk around? How did you sample them? Or did you attempt to survey *all* of them (which would be a census, not a sample, for that particular market)? Similar questions regarding the household interview --- how did you find the households? How you did you sample, once you had a list of some sort? You are not even attempting to describing your sampling techniques in anything close to a formal manner, and to my mind, this really damages the credibility of the paper and the results. Only for Zambia and Zimbabwe do you mention your sampling frame and your sampling strategy, and for Zimbabwe more detail is needed on how the snowball sampling was performed. | We have provided more information on how individuals were selected in each country in the Supplement, particularly Kenya and Ghana. For Tanzania, we have made it more clear that enumerators walked around the market and asked adult males and females if they had time to participate in a survey and that we did not attempt to survey all market participants (markets are very large 500-1000 people). We have also included more details on how household based interviews were conducted (i.e., the household head was available at the house) and how houses were initially identified (a combination of available records, walking around, and word of mouth). In general, we take the reviewers point that we need to be more explicit that we are not conducting representative sampling at the country level, which we believe may be an artifact of our title (“survey across countries”). We have made sure to specific that our study results cannot be said to be representative of countries in Lines 257-261. |
| **You give no information on power calculations**. This, of course is related. For instance, you decided to go with 200 people total in the 4 Zambaian camps. How did you choose that number? Ideally you would have done power calculations beforehand, to be sure that you could find meaningful statistics at the chosen sample size. If you did not do that, explain how you *did* pick those sizes. | Sample sizes were driven by a number of considerations including the preferences of governmental partners and the amount of resources available to FAO country team, logistical constraints such as availability of teams during periods of in-country conflict and safety considerations.  While our original power calculations suggested a target of 400+ households, because this was not feasible for any country, we have clarified in Lines 257-261 that our sample sizes were not based on power calculations and the consequences of this. |
| **Hand in hand with those questions is the question of “representativeness”** -- if I survey randomly within a certain district, my results may be said to be representative at the district level. It is not clear whether your results are representative for any market, district, etc. If not, that’s unfortunate, but it doesn’t kill your paper. However, you need to be clear. | We agree with the reviewer that we should be clearer about the representativeness of our samples across countries. We have included information in in Lines 257-261 specifying this. |
| **Who is being surveyed, in each household?** How do you choose your respondents? This is also commonly reported information for any survey. | We have provided more information on who fit the inclusion criteria in Lines 226-228. In brief, we restricted the survey to household heads and spouses of household heads where possible. |
| You need to be more clear about livestock included / not included in the survey. Are all livestock being represented or only birds? I think it might be only birds, as those are the only ones you speak about, but you never explicitly state it. This is particularly confusing for the Maasai since most of us will first think of cattle, w/ the Maasai. | We agree with the reviewer that this is helpful information to include and we have added this in Lines 218-221 in the revised manuscript. |
| You say that you chose those 3 production systems (layers, broilers, pastoralists) according to several listed factors. But the next sentence is not clear -- you list the concept but not the goal. That is, you chose according to what objective, for those variables? You wanted to choose production systems that made a significant contribution to GDP? (Presumably.) That were projected to grow significantly in future? And what about the patterns of AMU? What sort of pattern were you looking for? You should make it clear what your objectives were, when choosing these production systems. You should also explain why you chose those production systems in THOSE countries. Why Zambia, not Uganda? Why pastoralists in Tanzania rather than Ethiopia? | Thank you, we agree and have rewritten this section to clearly state the goals of our selection criteria. As for production systems, please see above (our first reply to Reviewer 8) for the reasoning behind country selection. |
| Where the KAP surveys identical across countries/production systems? If so, why would you do this? (Surely, for instance, some different questions are relevant to pastoralists than to folks owning chickens.) If not, how were they different across contexts? | The KAP survey was not identical across countries given the need to slightly tailor the questions to the production system and country context / language of delivery, but we can see how a reader would consider this the case. We have made this clear in Lines 221-225 as well as outlining where surveys were similar and different. |
| I do not understand why you are summarizing figures/statistics by country, rather than by production system, in the results. You chose your locations by production system. And your results are far from representative for any given country. So statements like “Ghana(ian) farmers kept the largest flocks” or “Kenya(n) farms had a median of 700 birds” seems misleading to me. First of all, you cannot say anything about “Ghanaian farmers” or “Kenyan farmers” in general. Second of all, isn’t the whole point to examine different production systems? So if anything, why not talk about farms keeping broilers vs. farms keeping layers vs. pastoralist farms? Are systems similar between Kenya and Ghana, given that you were targeting farmers keeping layers? It looks like not, from your results. | We agree that we would need to dramatically increase our sample size in order to make general statements about “Kenyan farmers” or “Ghanaian farmers”. We have made it more clear that when we say “Ghanaian farmers” we mean “Ghanian farmers interviewed in this survey”. In order to limit repeating this statement every time we refer to a farmer we put a note in Lines 259-261 for readers.  We focus our discussion on country-based descriptive statistics because variables entered into the model are country-level measures.  We also agree that it would be useful to compare and contrast production systems, aggregating stakeholder groups across countries together, and although this is outside the scope of this particular paper, indeed the project is continuing with plans to do exactly this. |
| I do not understand why you are pooling all of your data, in analysis, across countries and/or production systems. Relationships might differ significantly across contexts, so it seem imperative that you first present disaggregated analysis before pooling data. | We agree that our results could differ within countries. Our primary aim was to assess correlates of AMU and AMR across productions systems. Nevertheless, we recognize that differences may exist across countries, particularly due to sampling considerations. We attempt to control for some of differences by including country dummy in our models. The purpose of this project is multi-fold, and while this initial paper is a first-look at what is happening in an African context using select production systems and subsampled populations to get a handle on key factors driving antimicrobial use practices, the project is continuing with the intention of individual countries working on more narrowly focused country-specific investigations which will help to inform country-specific national action plans on antimicrobial resistance. |
| Ordinary least squares should not technically be applied to a count (rather than continuous) outcome variable. It is likely that using a more appropriate model will not make any difference, but you should still switch to a model that is appropriate for count data. Many websites and textbooks survey such models, e.g.: <https://stats.idre.ucla.edu/stata/seminars/regression-models-with-count-data/> | As the number of questions asked for each KAP domain was limited (7-10), our KAP outcome variables do take on a limited number of integer values and so we agree with the reviewer that the outcome can be considered count. However, after looking at the data, we decided on an OLS approach based upon a number of considerations: 1) the mean and variance of our outcome variables do not suggest poisson as the best modeling approach (knowledge mean=55, var=.07, attitudes mean = .62, var=.04, practices mean= .75, var =.02, 2); 2) there were no excess of zeros in the data (no zeros at all) meaning a zero-inflated model was not appropriate; and, 3) the sample size for pastoralist models (<200) ensures it is risky to examine using count models. We are also careful to ensure our models meet the assumptions of OLS and provide these tests in the Methods and Appendix. |
| **More importantly, it is not clear what is learned by these “total” K, A, P variables that you use in regression.** Knowledge is perhaps understandable --- it would seem that the higher this count variable is, the more knowledgeable the farmer is about antimicrobial medicines. But the Attitudes questions are not clear --- what it the “right” answer to each of these questions? If you have a high vs. low score, what does this say about your “attitude” towards antimicrobial medicine? If you cannot clearly state that a high score means this, and a low score means that, then it is not appropriate to use this variable as an outcome variable in a regression. Precisely the same point applies to practices -- I have no idea, from those questions, what it means if a farmer has a higher or lower practice count.  **If you cannot give an intuitive meaning behind those totals, then you should not use them.** Simply summarizing your survey information, by question, across contexts, is fine. It would be better not to use an index variable (the K, A, P totals) if it is not meaningful. | The conceptual framework of aggregating determinants around the three domains of knowledge, attitude and practices is very common in all research fields dealing with behavior questions. Our decision to label knowledge, attitudes, or practices as “right” was based on technical expert knowledge of the knowledge, attitudes, and practices that may give rise to the emergence, transmission, and maintenance of antimicrobial resistance. This was the “outside benchmark” that we used to assign whether reported responses were correct or incorrect. We do, however, agree with the reviewer that we could better define what we mean by our scores so that the underlying assumption is better expressed. With that in mind, we have added this information in the Introduction, Methods, and in Table 1  Briefly,  Knowledge score = level of understanding of AMR, antibiotics, residues, dosage regimes, vaccines.  Attitudes score = level of sensitivity about risks of AMU and appropriate use of antibiotics and alternatives (vaccines).  Practice score = implementation of actions promoting prudent AMU and which help to prevent the emergence (and spread) of AMR. |
| Your results and discussion could be better organized. The findings and statistics are all interesting, but it’s not always clear what holds each paragraph together, and in general I find it hard to read these sections and keep in mind the “highlights” or the most important findings. It feels too much like a list. | Thank you, we appreciate the feedback and have revised the discussion section accordingly. |
| Personally, I could do with many fewer acronyms. For instance, I do not see the point of using HMIC and HIC, and I think the use of KAP really hurts the readability of the paper. Even AMU vs AMR is confusing. This paper would be much easier to read if the number of acronyms was reduced. | We have limited the use of abbreviations to only those most commonly used in the literature (i.e., AMR, LMICs, and KAP), and have more clearly defined them. |
| Thematic analysis of WHAT qualitative data was used to develop the KAP survey? The source of qualitative data, and which countries and production systems were represented in this qualitative data, should be stated in the main manuscript. | We have been more specific on the types of qualitative data that was used to develop the KAP survey and their provenience in Lines 215-217. |
| Similarly, where were the survey pilots conducted? In each location? You need to be more specific about such details; the manuscript has many such details that are lacking. | Thank you, we have added more details on the locations of survey pilots. |
| You have a “Figure XXX” in the supplementary material on locations, under Tanzania. | We thank the reviewer for noticing this error. We have updated the supplement with the correct Figure number. |
