# Supplementary material for: Towards a bottom-up understanding of antimicrobial use and resistance on the farm: A knowledge, attitudes, and practices survey across livestock systems in five African countries
Source: PLoS One. 2020 Jan 24;15(1):e0220274. doi: 10.1371/journal.pone.0220274 (PMC6980545; doi:10.1371/journal.pone.0220274)
Supplement: Attachment — Submitted filename: LastPoints.docx [file pone.0220274.s007.docx]

Dear authors,

This version looks much better. A few minor things:

I think you should explicitly state, in the paragraph that begins on line 175, that the sampling was not representative of any given geographic region.

You say explicitly to two reviewers that you are controlling for a series of education dummies in your regressions, but Table 3 implies that you are including a categorical variable for education (values 0/1/2/3) in your regressions, which is not valid. You need to control for three dummies instead -- for primary, secondary, and tertiary, say. (This leaves “no education” as the omitted category.)

What you are doing w/ dropping “high influence” data point is considered old-fashioned and very poor practice in economics and statistics. But I’m satisfied that you say it doesn’t make a difference either way.

My only other comment is that there are still a number of small typos and (to my eye) grammatical problems, scattered throughout the manuscript. I did not take the time to list them comprehensively, but here are a few items that jumped out…

**Line 52:** Wrong use of semi-colon, bad use of parens. Should be:

…antimicrobial resistance (AMR), the phenomenon where microbes [‘germs’] acquire the ability to tolerate one or more drugs we rely on to treat microbial infections. [1–3].

**Line 64:** Need space between [7] and next word.

**Line 84**, the comma before constrains needs to be deleted. Should be:

Within most LMICs, the combined realities of underfunded veterinary healthcare systems and limited regulatory capacities constrain efforts to promote…

**Line 95** should instead have:

… the ratio of veterinarians to livestock is about 20 times lower **than** **that of** high-income countries of…

**Line 117** again wrong use of semi-colon, parens. Should be:

periods [27–36], the period of time before slaughter when treatments for the animal must cease in 118  order to effectively eliminate them from the animal's system.

**Line 413:** Should be “seventy percent of…” rather than “seventy% of”

**Table 6:** Not sure why variable is listed as treatment_failure instead of simply “treatment failure”
