# Supplementary material for: Towards a bottom-up understanding of antimicrobial use and resistance on the farm: A knowledge, attitudes, and practices survey across livestock systems in five African countries
Source: PLoS One. 2020 Jan 24;15(1):e0220274. doi: 10.1371/journal.pone.0220274 (PMC6980545; doi:10.1371/journal.pone.0220274)
Supplement: Attachment — Submitted filename: Rebuttal letter.docx [file pone.0220274.s008.docx]

| **Reviewer # 3** | **Response** |
| --- | --- |
| The first one, which is relatively minor, has to do with the definition of the education variable. Its description in Table 3 doesn’t correspond to that of three dummy variables. Please correct accordingly | Thank you. We have changed the definition of the education variable and made it more explicit how we entered education into the models. |
| The second one is more substantial and has to do with the process of backward elimination of variables whose coefficients do not reach a significance level of 5%. Why that level and not, say, a 10%? Can you provide some references for the use of this econometric procedure? If collinearity is not a problem, why would you take out variables out of the model even if they are not statistically significant?    Finally, why would you perform the backward elimination from 3 models: one controlling for sociodemographic variables exclusively, another one including only on-farm dynamics and a final one for sources of information? My previous concern about omitted variables bias was that THE model (ONE model, not three) should include ALL the potentially important (and potentially correlated) variables. | As we were building off significant results from earlier models, our approach to the backward elimination was to retain only those variables that were significant in those models. However, we take the reviewers point that if collinearity is not a problem, then we should not be overly concerned.  One of the reasons for taking the backwards approach due to the small sample size of the pastoralists group, where we wanted to replicate the pooled analysis but had under 200 observations. In addition, differences in predictor variables across models required separate models. For example, the amount of time engaged in a production system was not collected in Maasai given answers were always some version of “my entire life”. In short, while we needed to conduct two separate analyses, we didn’t have the sample size in pastoralists to rule out overfitting concerns.  We do take the reviewers point that omitted variable bias is a concern but we would like to retain the “three domain” analysis for simplifying the narrative. Due to concerns of omitted variable bias, we have included a full model in the Supplement and have removed the pooled stepwise regression from the paper. In the main manuscript, we discuss this new approach in the Methods (Lines 309-313) and then highlight where results of the full model differ from that of the full models. In most cases both the direction and size of effects were similar for domain-specific and pooled analyses. |
| **Reviewer # 7** |  |
| My first concern was that Education is NOT a continuous variable - yet I the table describing the Education variable they have not changed it. They have changed the regressions - so now what is in the table describing the data and the results are not consistent. | Thank you. We have changed the definition of the education variable and made it more explicit how we entered education into the models. |
| I was concerned that the graphs were difficult to follow - I find no new graphs so am unable to evaluate. | We did not change the graphs but provided better descriptions in the figure legends. We have provided more information in Lines 470-472. In addition, if the reviewer provides specifics on what it difficult to understand we can make the modifications. |
| **Reviewer # 8** |  |
| I think you should explicitly state, in the paragraph that begins on line 175, that the sampling was not representative of any given geographic region. | We agree that this needs to be made clear to the readers and have include this. |
| You say explicitly to two reviewers that you are controlling for a series of education dummies in your regressions, but Table 3 implies that you are including a categorical variable for education (values 0/1/2/3) in your regressions, which is not valid. You need to control for three dummies instead -- for primary, secondary, and tertiary, say. (This leaves “no education” as the omitted category.) | In our models, we do include three dummies (Primary Education, Secondary Education, Tertiary Education) and the no education was the omitted variable. We have made this clearer in Table 3. That is, we provide both how we defined education and how we entered it into models. |
| What you are doing w/ dropping “high influence” data point is considered old-fashioned and very poor practice in economics and statistics. But I’m satisfied that you say it doesn’t make a difference either way. | Thank you for this advice. While we knew that dropping of outliers was not recommended without a good reason, we did not realize this was an old practice. Thankfully, as the reviewer points out, inclusion and exclusion of these points do not impact model interpretation. |
| **Line 52:** Wrong use of semi-colon, bad use of parens. Should be:  …antimicrobial resistance (AMR), the phenomenon where microbes [‘germs’] acquire the ability to tolerate one or more drugs we rely on to treat microbial infections. [1–3]. | Thank you. Fixed |
| **Line 64:** Need space between [7] and next word. | Fixed |
| **Line 84**, the comma before constrains needs to be deleted. Should be:  Within most LMICs, the combined realities of underfunded veterinary healthcare systems and limited regulatory capacities constrain efforts to promote… | Fixed |
| **Line 95** should instead have:  … the ratio of veterinarians to livestock is about 20 times lower **than** **that of** high-income countries of… | Fixed |
| **Line 117** again wrong use of semi-colon, parens. Should be:  periods [27–36], the period of time before slaughter when treatments for the animal must cease in 118  order to effectively eliminate them from the animal's system. | Fixed |
| **Line 413:** Should be “seventy percent of…” rather than “seventy% of” | Fixed |
| **Table 6:** Not sure why variable is listed as treatment_failure instead of simply “treatment failure” | Fixed |
|  |  |
